# Mannose-6-Phosphate-Tagged Liposomes Exhibit Increased Transcytosis Across Human Blood–Brain Barrier Model

**DOI:** 10.3390/pharmaceutics18050619

**Published:** 2026-05-19

**Authors:** Margarita C. Dinamarca, Boris Sevarika, Scott McNeil

**Affiliations:** Nanopharmaceutical and Regulatory Science Group, Department of Pharmaceutical Sciences, University of Basel, Klingelbergstrasse 50, 4056 Basel, Switzerland; boris.sevarika@unibas.ch (B.S.); scott.mcneil@unibas.ch (S.M.)

**Keywords:** mannose-6-phosphate, liposomes, blood–brain barrier, human-induced pluripotent stem cells, transcytosis, CNS-targeted drug delivery

## Abstract

**Background/Objectives:** The blood–brain barrier (BBB) presents a major challenge for delivering therapeutics to the central nervous system (CNS) due to its highly selective permeability. Human brain microvascular endothelial cells (hBMECs), the principal cellular component of the BBB, tightly regulate molecular transport and restrict the entry of many CNS-targeted therapies. Lipid-based nanoparticles have emerged as promising carriers for BBB transport because of their biocompatibility, tunable surface properties, and cargo encapsulation capabilities. One strategy to enhance nanoparticle transport involves surface functionalization with ligands that exploit endogenous transcytosis pathways. Mannose-6-phosphate (M6P), a glycan implicated in the brain entry of certain proteins and viruses, represents a potential targeting ligand for this purpose. **Methods:** In this study, we established a physiologically relevant in vitro BBB model using human-induced pluripotent stem cell-derived brain microvascular endothelial cells (hiPSC-BMECs) to evaluate M6P-functionalized liposomes for BBB transport. Fluorophore-labeled liposomes were used to monitor nanoparticle uptake and transcytosis. **Results:** M6P-functionalized liposomes exhibited significantly enhanced uptake in hiPSC-BMECs compared with non-functionalized control liposomes. Pharmacological inhibition studies supported the involvement of a clathrin-sensitive endocytic pathway. Transcytosis assays demonstrated enhanced BBB crossing of M6P-functionalized liposomes, with transport increasing according to ligand density and reaching approximately 55% of the transport observed for transferrin under the same experimental conditions. Following transcytosis, intact M6P-functionalized liposomes showed significantly higher uptake by downstream hiPSC-derived neurons and astrocytoma cells compared with control formulations. **Conclusions:** Together, these findings support M6P-functionalization as a promising strategy to enhance liposome uptake and transcytosis across a human-relevant in vitro BBB model. This work provides a proof-of-concept framework for the development and optimization of glycan-functionalized nanocarriers for CNS-directed delivery.

## 1. Introduction

Treating central nervous system (CNS) diseases remains one of the most difficult challenges in medicine, primarily due to the presence of the blood–brain barrier (BBB). This highly selective barrier, composed mainly of human brain microvascular endothelial cells (hBMECs) along with astrocytes and pericytes, strictly regulates the exchange of substances between the bloodstream and brain tissue [1,2]. While vital for maintaining neural homeostasis and protecting the brain from toxins and pathogens, the BBB also severely limits the entry of therapeutics. It is estimated that over 98% of small-molecule drugs and nearly all large biologics fail to cross the BBB in therapeutically meaningful amounts [2]. As a result, many potentially effective CNS therapies reach their target sites in the brain in insignificant amounts.

Liposomes, a subset of lipid nanoparticles, are a well-established platform for delivering a range of therapeutic agents, including nucleic acids and small molecules. Their clinical success, most notably in mRNA vaccines, has demonstrated their potential as safe and versatile delivery vehicles [3,4]. Lipid nanoparticles offer distinct advantages such as biocompatibility, protection of sensitive cargo, and the ability to be functionalized for targeted delivery. However, despite their systemic delivery capabilities, conventional liposomes show poor accumulation in the brain, primarily due to the restrictive nature of the BBB and their lack of inherent targeting mechanisms [5,6]. This limitation remains a significant bottleneck in applying liposome technology to neurological diseases.

One of the most widely studied approaches for CNS targeting has been the use of transferrin, a glycoprotein that binds to the transferrin receptor, which is highly expressed on the luminal surface of brain endothelial cells. Transferrin-mediated transcytosis has become a gold-standard strategy for delivering therapeutics across the BBB due to its well-characterized mechanism and efficient transport capabilities [2,7,8]. Numerous studies have demonstrated that nanoparticles or biologics conjugated with transferrin or anti-transferrin receptor antibodies exhibit significantly enhanced brain uptake [8,9,10,11,12,13]. Despite its success, challenges such as receptor saturation and off-target effects have prompted ongoing research into alternative or complementary targeting ligands [7]. Nonetheless, transferrin remains a benchmark against which emerging CNS-targeting strategies are often compared.

Addressing this challenge requires the development of brain-targeted delivery strategies. One promising approach involves engineering liposomes with surface ligands that exploit endogenous transport systems at the BBB. Mannose-6-phosphate (M6P) plays a key role in cell biology as a ligand involved in intracellular trafficking, particularly in the targeting of lysosomal enzymes. The cation-independent mannose-6-phosphate receptor (CI-M6PR), which facilitates lysosomal enzyme trafficking, is expressed on several endothelial cell types, including those in the brain vasculature [14,15]. M6P ligand facilitates the entry of specific viruses, such as varicella-zoster virus, HIV-1, and rotavirus, into the brain. It also mediates the transport of proteins like renin, potentially via receptor-mediated transcytosis [16,17,18,19]. Functionalizing liposomes with M6P ligands may, therefore, be used as a strategy to increase the uptake and transcytosis across the BBB, offering a route for minimally disruptive, targeted delivery into the CNS.

To evaluate such strategies, physiologically relevant in vitro blood–brain barrier (BBB) models are essential. Traditional models, which often use immortalized cell lines or animal-derived endothelial cells, typically fail to recapitulate the tight junction integrity, transporter expression, and functional characteristics of the human BBB. In contrast, BBB models derived from human-induced pluripotent stem cells (hiPSCs) provide a more accurate and scalable platform for studying nanoparticle transport [18]. hiPSC-derived human brain microvascular endothelial cells (hiPSCs-BMECs) closely mimic in vivo BBB properties, forming a tight monolayer that expresses brain vasculature-specific markers, exhibits polarized transporter localization, maintains high transendothelial electrical resistance (TEER), and possesses functional efflux systems [19]. Moreover, these models enable patient-specific investigations, as hiPSCs can be generated from individual patients to produce hBMECs that retain the donor’s unique genetic and phenotypic characteristics. This allows researchers to model BBB properties in the context of specific genetic backgrounds or disease states, providing a platform to study variability in nanoparticle transport and therapeutic responses. They also support high-throughput screening, making them well-suited for assessing nanoparticle design and transport mechanisms [20,21,22,23].

However, early differentiation protocols, including those established by Lippmann et al. [20] and Hernando et al. [21], produce cells with limited endothelial fidelity. These cells often display epithelial-like properties, with high transendothelial electrical resistance (TEER) but suboptimal expression of key endothelial markers such as VE-cadherin (CDH5) and PECAM-1 (CD31). One major limitation of these classical approaches is the lack of activation of canonical Wnt/β-catenin signaling, a pathway known to be essential for BBB specification during central nervous system (CNS) development [22,23,24]. In vivo, Wnt ligands secreted by neural progenitors induce BBB properties in adjacent endothelial cells, promoting tight junction formation, transporter expression, and selective permeability. The omission of this signaling cue during in vitro differentiation can lead to incomplete or aberrant specification of iPSC-BMECs [22,23,24].

In this study, we incorporate activation of canonical Wnt/β-catenin signaling into classical differentiation protocols for hiPSC-derived human brain microvascular endothelial cells to establish a physiologically relevant in vitro blood–brain barrier model. This model was used to investigate the transcytosis efficiency of M6P-functionalized liposomes, which, in our previous studies, have been shown to exhibit enhanced uptake in various cell lines due to the presence of M6P on their surface [25]. Here, we demonstrate that M6P-functionalized liposomes show increased uptake in hiPSC-BMECs via a clathrin-dependent mechanism. Moreover, we observe enhanced transcytosis across hiPSC-BMECs, which is specific to the M6P ligand. Subsequent transcytosis assays demonstrated effective BBB crossing, with transport efficiency positively correlated with ligand density and reaching up to 55% of that observed with transferrin-mediated transcytosis. Following transcytosis, M6P liposomes delivered higher payloads to downstream hiPSC-derived neurons and astrocytoma cells compared to control formulations. By combining targeted nanocarrier engineering with a physiologically relevant human BBB model, our goal is to contribute to overcoming one of the most persistent challenges in neurotherapeutics: the effective transport of therapeutics across the BBB.

## 2. Materials and Methods

### 2.1. Synthesis of M6P Ligand and Control Molecules

We synthesized DSPE-PEG-mannose-6-phosphate (M6PG), DSPE-PEG-sialic acid (SA), and DSPE-PEG-carboxylic acid (COOH) conjugates using a previously published protocol [25]. The identity and purity of the prepared molecules were confirmed using the UPLC-ELSD method [25].

### 2.2. Preparation of Liposomes

Liposomes were prepared using the thin-film hydration method. All developed formulations contained cholesterol (Cat. No. C8667, Sigma Aldrich, Burlington, MA, USA), unsaturated C18 phospholipid DOPC (Cat. No. 850375, Avanti Polar Lipids, Alabaster, AL, USA), and fluorescent marker DiO (Cat. No. 60011, Biotium, Fremont, CA, USA) or DiD (Cat. No. 60014, Biotium, Fremont, CA, USA). Synthesized lipid–PEG–ligand conjugates, DSPE-PEG8-M6P, DSPE-PEG8-SA, and DSPE-PEG8-COOH, and purchased ganglioside GM1 (Cat. No. 860094, Avanti Polar Lipids, Alabaster, AL, USA), were used for liposomes functionalization. DSPE-PEG8-OCH3 was used as a control (bp-22174, BroadPharm, San Diego, CA, USA). The overall concentration of the hydrophobic components in the final products was kept constant at 10 mM.

Lipid components were dissolved in methanol (Cat. No. P717.1, Carl Roth, BL, Switzerland), ethanol (Cat. No. 1HPH.1, Carl Roth, BL, Arlesheim, Switzerland), chloroform (Cat. No. 3313.1, Carl Roth, BL, Arlesheim, Switzerland), or mixtures thereof and mixed together. Organic solvent was removed using a rotary evaporator (50 °C, 150 mbar) to obtain thin lipid films, which were kept for an additional 2 h under vacuum (25 °C, 20 mbar) to remove residual solvents.

The dry films were hydrated using PBS (Cat. No. D8537, Sigma Aldrich, Burlington, MA, USA) to obtain the final lipid concentration of 10 mM. The resulting lipid suspension was then sonicated for 30 min at 35 °C at an ultrasound frequency of 37 kHz using an Elmasonic S 60 (H) sonicator (Elma Schmidbauer, Singen, Germany) and then extruded 10 times through a membrane with a pore size of 100 nm to optimize the size distribution (Cat. No. 610000, Avanti Polar Lipids, Alabaster, AL, USA).

### 2.3. Liposomes Characterization

The size and size distribution of prepared samples were measured using dynamic light scattering (DLS) and a Zetasizer Pro system (Malvern, Worcestershire, UK). The measurements were conducted at 25 °C, with an equilibration time of 120 s, using the backscattering detector. The attenuation factor was determined automatically (ZS Explorer, Malvern Panalytical, Worcestershire, UK). The sample was prepared by diluting the prepared liposomes in PBS (20-fold dilution). The size measurements were performed in three runs. A fluorescence filter was applied to mitigate any interference from the fluorescent dye. The scattering intensity-based size distributions are shown.

To determine the zeta potential of liposomes formulations, we used electrophoresis light scattering (ELS) and a Zetasizer Pro system (Malvern, Worcestershire, UK). The sample was prepared by diluting the original 10 mM liposomes stock with 10 mM NaCl solution (20-fold dilution). The instrument software (4.2.1 version, ZS Explorer, Malvern Panalytical, Worcestershire, UK) computed the ideal voltage and attenuation factor for each measurement. The equilibration time of 10 s was used. The measurements were conducted at 25 °C. The average zeta potential is reported.

The fluorescence of all formulations used to create each independent set of data was determined with a TECAN Infinite PRO 200 plate reader (Tecan Trading, Männedorf, Switzerland) to ensure the comparability of prepared formulations. The excitation wavelength was set according to the fluorescence marker used (488 for DiO and 640 for DiD) [25].

### 2.4. hiPSCs Maintenance and hiPSC-BMECs Differentiation

The hiPSC line used in this study was ChiPSC12, and it was a gift from Dr. Amandine Grimm (Department of Biomedicine, University of Basel). hiPSCs were cultured in Matrigel-coated dishes (according to the manufacturer’s recommendations, Cat. No. 354277, Corning, New York, NY, USA) in mTeSR Plus medium (Cat. No. 100-0276, STEMCELL Technologies, Vancouver, BC, Canada) with 1% Pen/Strep (100 units/mL of penicillin and 0.1 mg/mL streptomycin (Cat. No. 300300, Sigma Aldrich, Burlington, MA, USA)). When the confluence of hiPSCs was up to 80%, cells were passaged after a 5 min incubation at 37 °C with CTS™ Versene™ solution (Cat. No. A423910, Thermo Fisher Scientific, Waltham, MA, USA). Then, using a 5 mL pipette, cells were gently dissociated and passaged as 1:3–1:8 split ratios onto Matrigel-coated six-well plates with the mTeSR Plus medium supplemented with 10 µM ROCK inhibitor Y27632 (Cat. No. 72307, STEMCELL Technologies, Vancouver, BC, Canada) for 24 h. Afterwards, the cells were kept with the mTSR Plus medium. For BMEC differentiation, we used hiPSCs up to passage 45 [21].

For hiPSC-BMECs differentiation, we followed the protocol by Hernando et al., with slight modifications [21]. hiPSCs were maintained in mTeSR Plus media as described above. Four days before differentiation induction (D-4), cells were washed with Dulbecco’s phosphate-buffered saline (DPBS, Cat. No. D8537, Sigma Aldrich, Burlington, MA, USA); 500 µL of TrypLE™ express enzyme (Cat. No. 12605036, Thermo Fisher Scientific, Waltham, MA, USA) was added to each well and passaged after a 5 min incubation at 37 °C. Next, cells were diluted at a ratio of 1:5 in mTeSR media, centrifuged for 5 min at 400× *g*, and resuspended in mTeSR media supplemented with ROCK inhibitor Y27632 (10 µM).

HiPSCs were seeded onto Matrigel-coated six-well plates at 3.1 K/cm^2^ cell density. To start the differentiation (D0), mTeSR was removed and changed to TeSR™-E6 media (Cat. No. 05946, STEMCELL Technologies, Vancouver, BC, Canada). We repeated the procedure daily for four days (D0–D3). At D2, the medium was changed to E6 medium supplemented with 6 µM ChIR to promote mesoderm induction during 24 h. Then, the medium was switched to Human Endothelial Serum-Free Medium (hESFM, Cat. No. 11111044, Thermo Fisher Scientific, Waltham, MA, USA) supplemented with 1X B27, 20 ng/mL bFGF (Cat. No. 100-18B-50UG, Thermo Fisher Scientific, Waltham, MA, USA), and 10 µM retinoic acid (RA, Cat. No. 044540.77, Thermo Fisher Scientific, Waltham, MA, USA).

Cells were maintained in this medium for two consecutive days without a medium change. After those two days, the medium was removed, and the wells were washed with DPBS and incubated with TrypLE for 10 to 15 min at 37 °C until a single-cell suspension was formed. The cells were then subcultured onto 6.5 mm Transwell^®^ filters with a 3 µm pore size (Cat. No. 3452, Corning, New York, NY, USA), previously coated with a mixture of 400 μg/mL collagen IV (Cat. No. C5533, Sigma Aldrich, Burlington, MA, USA) and 100 μg/mL fibronectin (Cat. No. F1141, Sigma Aldrich, Burlington, MA, USA) in water; 1 × 10^6^ cells were seeded on each transwell membrane with hESFM supplemented with 1X B27, 20 ng/mL bFGF, 10 µM RA, and 6 µM ChIR for 24 h. Then, the medium was changed to hESFM media with B27 without bFGF, RA, and ChIR.

### 2.5. TEER Measurement

TEER was measured using STX2 chopstick electrodes and an EVOM2 voltohmmeter (World Precision Instruments, Friedberg, Germany) 48–72 h after subculturing. TEER values show the mean of independent differentiations, and all values were corrected for the resistance of an empty, coated Transwell filter. TEER was used as a monolayer tightening reference, with TEER below 3000 Ω·cm^2^ at 72 h post-subculture omitted for follow-up experiments.

### 2.6. Astrocytoma Cells

Astrocytoma cell line 1321 N1 astrocytoma (Cat. No. 86030402, Sigma Aldrich, Burlington, MA, USA) was cultured in high glucose DMEM medium supplemented with GlutaMAX™ and phenol red (Cat. No. 11594446, Thermo Fisher Scientific, Waltham, MA, USA), 10% heat-inactivated fetal bovine serum (FBS, Cat. No. 11550356, Thermo Fisher Scientific, Waltham, MA, USA), and 1% Pen/Strep.

### 2.7. hiPSC Differentiation to Neurons

The iPSC line used for neuronal differentiation was a gift from Dr. Eline Pecho-Vrieseling. The line was generated from healthy adult human dermal fibroblast lines from a 32-year-old female (Cat. No. C-013-5C, Invitrogen, Waltham, MA, USA) and has an inducible Ngn2 gene under a doxycycline promoter. The neuronal differentiation protocol is described in Russell et al. [26] with smaller modifications. Briefly, hiPS cells were plated on Matrigel in a proliferation medium composed of DMEM/F12 with GlutaMAX™ (Cat. No. 10565018, Thermo Fisher Scientific, Waltham, MA, USA) supplemented with 1X B-27™ supplement (Cat. No. 100-18B-50UG, Thermo Fisher Scientific, Waltham, MA, USA) and 1x N-2 supplement (Ca. No. 17502048, Thermo Fisher Scientific, Waltham, MA, USA), 1% Pen/Strep, 10 ng/mL hEGF (Cat. No. PHG0311, Thermo Fisher Scientific, Waltham, MA, USA), 10 ng/mL hFGF (Cat. No. RFGFB50, Invitrogen, Waltham, MA, USA), and 10 µM Rock inhibitor (RI) for 1 day and 1 µg/mL doxycycline for 3 days. The progenitors were kept frozen in a Cryostor freezing medium (Ca. No. 100-1061, STEMCELL Technologies, Vancouver, BC, Canada) or replated for immediate experiments. Progenitors were thawed and seeded on top of the cover glass in a 12-well plate format. A total of 3.0 × 10^5^ cells were plated in a neuronal differentiation medium composed of the Neurobasal™ medium (Cat. No. 21103049, Thermo Fisher Scientific, Waltham, MA, USA), B27, N2 supplements, 1% Pen/Strep/Glutamax supplemented with 10 ng/mL BDNF (Cat. No. 248-BD, R&D Systems, Minneapolis, MN, USA) and 10 ng/mL GDNF (Cat. No. 212-GD, R&D Systems, Minneapolis, MN, USA). Starting from day 2 of the co-culture, medium change was done every other day.

### 2.8. Western Blot and Immunofluorescence

hiPSC-BMECs and iPSCs were harvested, washed twice with ice-cold PBS (Cat. No. D8537, Sigma Aldrich), and lysed in a RIPA Lysis and Extraction Buffer (Cat. No. 89900, Thermo Fisher Scientific, Waltham, MA, USA) supplemented with a cOmplete™ EDTA-free protease inhibitor mixture (Cat. No. 11836170001, Sigma Aldrich, Burlington, MA, USA). Lysates were incubated on ice for 15 min and cleared via centrifugation (10,000× *g*) for 10 min at 4 °C. Supernatants were collected, and the protein concentration was determined using a BCA assay kit (Cat. No. 23225, Thermo Fisher Scientific, Waltham, MA, USA). Lysates were resolved using standard SDS-PAGE gels (Mini-PROTEAN TGX Stain-Free Precast Gels 4–20%, Cat. No. 4568091, Bio Rad, Hercules, CA, USA), and after blocking, blots were incubated with primary antibodies overnight at 4 °C. After washing, the blots were incubated with secondary antibodies and visualized using a SuperSignal™ Femto (Cat. No. 34095, Thermo Fisher Scientific, Waltham, MA, USA) in an Odyssey^®^ Imager C (LI-COR, Lincoln, NE, USA). Quantification of the Western blot was done using Fiji Image J software v1.38e (NIH, Bethesda, MD, USA) and an integrated density plugin. The values were normalized to the expression of the loading control.

For immunostaining, hiPSC-BMECs on glass coverslips (24-well plate format, 0.5 × 10^6^ cells/well) were fixed for 10 min in 4% PFA (Cat. No. 1.00496, Sigma Aldrich, Burlington, MA, USA) at room temperature, permeabilized with PBS containing 1 mM MgCl_2_, 0.1 mM CaCl_2_, and 0.1% Triton™ X-100 (Cat. No. T8787, Sigma Aldrich, Burlington, MA, USA), blocked with 5% BSA (Cat. No. 10711454001, Sigma Aldrich, Burlington, MA, USA) in PBS, and labeled with primary antibodies in PBS with 5% BSA overnight at 4 °C and secondary antibodies for 45 min at room temperature. PBS washing was performed after each antibody incubation. Coverslips were mounted in ProLong™ Diamond Antifade Mountant (Cat. No. P36965, Invitrogen, Waltham, MA, USA).

Anti-GluR1, Bassoon, and MAP2 were purchased from Synaptic Systems. Anti-EpCAM, anti-VE Cadherin, anti-M6PR-CI, anti-P-Glycoprotein, anti-Tfr receptor, anti- RPL36, anti-GLUT1, Phalloidin, anti-Occludin, and anti-ZO-1 antibodies were purchased from Abcam (Cambridge, UK). All secondary antibodies were from Abcam (Cambridge, UK).

### 2.9. Particle Uptake Experiments

Uptake experiments were conducted in cells cultured on cover glass in 24-well plates coated with a mixture of 400 μg/mL collagen IV (Cat. No. C5533, Sigma Aldrich, Burlington, MA, USA) and 100 μg/mL fibronectin (Cat. No. F1141, Sigma Aldrich, Burlington, MA, USA) in water; 0.5 × 106 cells were seeded in each well with hESFM (Cat. No. 11111044, Thermo Fisher Scientific, Waltham, MA, USA) supplemented with the B-27™ supplement (Cat. No. 17504044, Thermo Fisher Scientific, Waltham, MA, USA), 20 ng/mL bFGF (Cat. No. 100-18B-50UG, Thermo Fisher Scientific, Waltham, MA, USA), 10 μM retinoic acid (Cat. No. R2625, Sigma Aldrich, Burlington, MA, USA), and 3 μM CHIR99021 (Cat. No. 72052, STEMCELL Technologies, Vancouver, BC, Canada) for 24 h. Then, the medium was changed to hESFM (Cat. No. 11111044, Fisher Scientific, Waltham, MA, USA) with the B-27™ supplement (Cat. No. 17504044, Thermo Fisher Scientific, Waltham, MA, USA) without bFGF, retinoic acid, and CHIR99021. On the day of the experiment, liposomes were diluted in the FluoroBrite™ DMEM medium (Cat. No. A1896701, Thermo Fisher Scientific, Waltham, MA, USA) supplemented with GlutaMAX™ (Cat. No. 35050061, Thermo Fisher Scientific, Waltham, MA, USA), penicillin–streptomycin (Cat. No. 300300, Sigma Aldrich, Burlington, MA, USA), and the B-27™ supplement (Cat. No. 17504044, Thermo Fisher Scientific, Waltham, MA, USA).

Cells were incubated for the specific time point described in the experiment with the liposome formulations at 37 °C under a 5% CO_2_-humidified atmosphere on an orbital shaker (50 rpm). Next, the cells were washed three times with PBS to remove unbound liposomes. To visualize cells, the cell membrane was stained with CellMask (1:1000 dilution in PBS, 10 min at room temperature, Cat. No. H32721, Thermo Fisher Scientific, Waltham, MA, USA) or SPY555-actin (according to the manufacturer’s instructions, Cat. No. SC301, Spirochrome, Zürich, Switzerland). Nuclei were stained with Hoechst 33342 (Cat. No. H3570, Thermo Fisher Scientific, Waltham, MA, USA) or DAPI (Cat. No. D1306, Thermo Fisher Scientific, Waltham, MA, USA), and liposomes were directly visualized by DiO or DiD fluorescence. After live cell staining, cells were washed three times with PBS, fixed for 10 min in 4% PFA (Cat. No. 28908, Invitrogen) at room temperature, and mounted on glass slides in ProLong™ (Cat. No. P36930, Invitrogen, Waltham, MA, USA).

### 2.10. Image Acquisition and Analysis

Fluorescence signals were imaged with a Zeiss LSM-700 system with a Plan-Apochromat 40×/NA 1.30 oil DIC objective (Carl Zeiss, Oberkochen, Germany) and operated with ZEN 2010 software Version 6.0 (Carl Zeiss, Oberkochen, Germany). All images were acquired with identical microscope settings within individual experiments. Saturation was avoided by using image acquisition software (ZEN 2010 software (Carl Zeiss, Oberkochen, Germany) to monitor intensity values. For any image adjustment, identical settings were always applied to all cells. For quantification, values were averaged over multiple cells from at least three independent cultures and liposomes preparation.

Quantification of images was done using Fiji Image J software v1.38e software (NIH, Bethesda, MD, USA). Images were subtracted from the background, and after setting an automated threshold, the “Analyze Particles” plugin was used to determine the number, size and MFI of liposomes.

### 2.11. Endocytosis Inhibitor and Liposomes Uptake Measured by Flow Cytometry

hiPSC-BMECs were cultured in 24-well plates coated with a mixture of 400 μg/mL collagen IV (Cat. No. C5533, Sigma Aldrich, Burlington, MA, USA) and 100 μg/mL fibronectin (Cat. No. F1141, Sigma Aldrich, Burlington, MA, USA) in water. A total of 0.5 × 10^6^ cells were seeded in each well with hESFM (Cat. No. 11111044, Fisher Scientific, Waltham, MA, USA) supplemented with the B-27™ supplement (Cat. No. 17504044, Fisher Scientific), penicillin–streptomycin (Cat. No. 300300, Sigma Aldrich, Burlington, MA, USA), 20 ng/mL bFGF (Cat. No. 100-18B-50UG, Fisher Scientific, Waltham, MA, USA), 10 μM retinoic acid (Cat. No. R2625, Sigma Aldrich, Burlington, MA, USA), and 3 μM CHIR99021 (Cat. No. 72052, STEMCELL Technologies, Vancouver, BC, Canada) for 24 h. Then, the medium was changed to hESFM (Cat. No. 11111044, Fisher Scientific, Waltham, MA, USA) with the B-27™ supplement (Cat. No. 17504044, Thermo Fisher Scientific, Waltham, MA, USA) without bFGF, retinoic acid, or CHIR99021.

On the day of the experiment, the cells were preincubated with 25 μM Pitstop 2 (Cat. No. SML1169, Sigma Aldrich, Burlington, MA, USA) for 15 min, followed by liposomes treatment for a further 2 h at a total lipid concentration of 500 μM in the continued presence of the inhibitors in the FluoroBrite™ DMEM medium (Cat. No. A1896701, Fisher Scientific, Waltham, MA, USA) supplemented with GlutaMAX™ (Cat. No. 35050061, Fisher Scientific, Waltham, MA, USA), penicillin and streptomycin (Cat. No. 300300, Sigma Aldrich, Burlington, MA, USA), and the B-27™ supplement (Cat. No. 17504044, Fisher Scientific, Waltham, MA, USA) at 37 °C under a 5% CO_2_-humidified atmosphere on an orbital shaker (50 rpm). Cells were then washed three times with PBS (Cat. No. D8537, Sigma Aldrich, Burlington, MA, USA) to remove unbound liposomes.

After washing, hiPSC-BMECs were incubated for 5 min in phenol red-free trypsin/EDTA solution (0.25% trypsin and 0.2% EDTA, Cat. No. 25200056, Thermo Fisher Scientific, Waltham, MA, USA). The resulting cell suspension was filtered through a 70 µm nylon mesh cell strainer (Cat. No. 352350, Corning, New York, NY, USA) to remove aggregates. Cells were stained for 5 min with propidium iodide (final concentration 1.2 µg/mL, Cat. No. P4170, Sigma Aldrich, Burlington, MA, USA). Flow cytometry was performed immediately afterward using a BD LSR Fortessa™ flow cytometer (BD Bioscience, Heidelberg, Germany).

Forward and side scattering detectors were used to discriminate between single cells, cell debris, or aggregates. The discrimination between live and dead cells was based on the intensity of the propidium iodide signal, and liposome uptake was determined by DiO fluorescence intensity. For propidium iodide, the excitation wavelength was 561 nm, and emission was detected using a 610/20 nm filter. For DiO, the wavelength of 488 nm was set for excitation, and the fluorescence was measured using a 512/25 nm filter. Mean fluorescence values from at least three parallel wells were averaged to quantify particle uptake, and the cell autofluorescence was subtracted from the fluorescence values.

### 2.12. Transcytosis Assay

Transcytosis assays were performed using human-induced pluripotent stem cell-derived brain microvascular endothelial cells (hiPSC-BMECs) seeded on 12-well Transwell^®^ inserts with 3 μm pore size (Cat. No. 3402, Corning, New York, NY, USA). Cells were cultured at a density of 1 × 10^6^ cells per insert and allowed to mature until tight junctions were established. Barrier integrity was verified by transendothelial electrical resistance (TEER) measurements using an EVOM3 voltohmmeter (World Precision Instruments, Friedberg, Germany). Only monolayers exhibiting TEER values ≥ 3000 Ω·cm^2^ were included in downstream assays.

To further confirm the integrity of the barrier and rule out paracellular leakage, 70 kDa FITC-dextran (Cat. No. 46945, Thermo Fisher Scientific, Waltham, MA, USA) or Rhodamine-dextran (Cat. No. R9379, Sigma Aldrich, Burlington, MA, USA) was added to the apical compartment in select wells. Samples were collected from the basolateral compartment during or after 6 h incubation, and fluorescence intensity was quantified using a TECAN Infinite^®^ PRO 200 plate reader (Tecan Trading AG, Männedorf, Switzerland). Monolayers with detectable dextran leakage above background levels were excluded from analysis.

Before initiating the transcytosis assay, monolayers were equilibrated in an experimental medium consisting of FluoroBrite™ DMEM (Cat. No. A1896701, Fisher Scientific, Waltham, MA, USA) supplemented with GlutaMAX™ (Cat. No. 35050061, Fisher Scientific, Waltham, MA, USA), penicillin–streptomycin (Cat. No. 300300, Sigma Aldrich, USA), and the B-27™ supplement (Cat. No. 17504044, Fisher Scientific, Waltham, MA, USA). The plates were maintained under a 5% CO_2_-humidified atmosphere on an orbital shaker (50 rpm) for 30 min.

Fluorescently labeled liposomes, formulated with DiO (Cat. No. 60011, Biotium, Fremont, CA, USA) or DiD (Cat. No. 60014, Biotium, Fremont, CA, USA), were diluted in an experimental medium to a final lipid concentration of 100 μM and added to the apical chamber. In each experimental condition, 100 μL aliquots were collected from the basolateral chamber at designated time points (as described in the figure legends) and immediately replaced with 100 μL of fresh experimental medium to maintain a constant volume.

The fluorescence of transported liposomes was measured using a TECAN Infinite PRO 200 plate reader (Tecan Trading, Männedorf, Switzerland). Values were background-corrected by subtracting the fluorescence of the blank medium, and the amount of transported liposomes was calculated as a percentage of the initial input fluorescence (100%). All samples were measured in technical duplicates or triplicates. As a reference for receptor-mediated transcytosis, transferrin-Cy3 (Cat. No. T2872, Thermo Fisher Scientific, Waltham, MA, USA) was included in parallel wells and treated identically. Experiments were performed with at least three independent biological replicates and liposomes preparation unless otherwise indicated.

### 2.13. Nanoparticle Tracking Analysis

The integrity of liposomes was analyzed using a nanoparticle tracking analysis (NTA) system from ParticleMetrics (Inning am Ammersee, Germany). The COLOC5POSITIONS script was used, and the conditions were verified using 0.1 μm of TetraSpeck™ microspheres (Cat. No. T7279, Thermo Fisher Scientific, Waltham, MA, USA), fluorescent in dilutions 1:400 and 1:1000. Once the system was aligned, the sample channel was washed several times. At the end of the transcytosis experiment, a 5 µL aliquot was taken from the abluminal medium and diluted in H_2_O to a final dilution between 1:100 and 1:10,000 (Ct or M6P). Half of the volume was mixed with Triton™ X-100 (Cat. No. T8787, Sigma Aldrich, Burlington, MA, USA) to a final concentration of 0.1%, and 1 mL was injected to measure particle concentration in fluorescence and scattering mode.

### 2.14. Statistical Analysis

Data analysis was performed with GraphPad Prism version 8.0 (GraphPad Software, La Jolla, CA, USA). Individual data sets were tested for normality with the Shapiro–Wilk, D’Agostino and Pearson, or Kolmogorov–Smirnov test. The statistical significance of differences between groups was assessed by unpaired or paired two-tailed Student’s *t*-test, Mann–Whitney test, one-way ANOVA with Tukey post hoc test. Data are presented as mean ± standard error of the mean (s.e.m.). A Table with the raw data obtained in this study is available in Supplementary Materials.

## 3. Results and Discussion

### 3.1. Characterization of hiPSC-Derived BMEC

**Figure 1:**
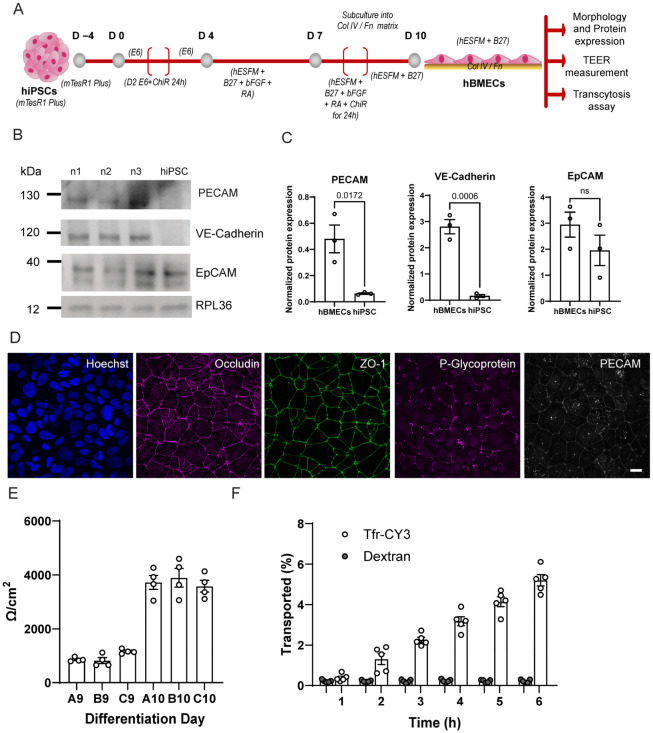
Differentiation of hiPSCs into brain microvascular endothelial-like cells (hiPSC-BMECs) and their characterization. (**A**) Schematic of the differentiation timeline from human-induced pluripotent stem cells (hiPSCs) into hiPSC-BMECs, showing key media changes and treatment conditions across days −4 to 10. (**B**) Western blot shows 3 different “n” values for differentiation and hiPSC, illustrating the expression of key adhesion proteins such as PECAM, VE-Cadherin, and EpCAM, together with loading control RPL36. (**C**) Western blot analysis and quantification of VE-Cadherin, PECAM, and EpCAM protein expression levels in hiPSCs and differentiated hiPSC-BMECs (10 days of differentiation). Data are shown as a normalized expression mean ± s.e.m.; statistical significance is indicated, ns = non-significant, Student’s *t*-test. Each point is an independent differentiation culture. (**D**) Immunofluorescence staining of hiPSC-BMECs at day 10 of differentiation for tight junction and endothelial markers: Hoechst (nuclei), Occludin, ZO-1, PECAM, and P-Glycoprotein. Bar = 10 µm. (**E**) Transendothelial electrical resistance (TEER) measurements of 3 independent cultures (A, C and C) over the course of differentiation indicate barrier formation. Day 10 was used for transcytosis assays. Each data point represents one transwell. (**F**) Quantification of dextran-FITC and transferrin-Cy3 transport across the hiPSC-BMECs monolayer on the transwell system over time (1 to 6 h), measured as a % of the initial fluorescence, corresponding to 1.25 µg/mL dextran-FITC (70 KDa) and 20 µg/mL Tfr-Cy3. Each data point represents one transwell from an independent differentiation culture. Data is shown as mean ± s.e.m.

Using CHIR99021, a well described and widely used GSK3 inhibitor [27,28], we incorporate activation of canonical Wnt/β-catenin signaling into classical hiPSC-derived human brain microvascular endothelial cells differentiation protocols [20,21,29,30]. We sought to slightly modify a protocol from Hernando et al. [21]. Our differentiation scheme is shown in Figure 1A, where 6 µM of CHIR99021 [31] was added during the differentiation process and after hiPSC-BMEC progenitors have formed. Incorporating CHIR99021 into differentiation protocols thus represents a critical refinement, addressing the limitations of earlier methods and enabling the generation of hiPSC-BMECs with improved endothelial characteristics and BBB functionality [31]. Differentiation of iPSCs into hBMECs was verified by the evaluation of the expression levels of endothelial (Platelet Endothelial Cell Adhesion Molecule-1 (PECAM) and Vascular Endothelial (VE-Cadherin)) and epithelial (Epithelial Cell Adhesion Molecule (EpCAM)) markers using Western blot. As shown in Figure 1B,C, the expression levels of the endothelial adhesion proteins PECAM and VE-Cadherin were significantly increased in hiPSC-BMECs compared to undifferentiated cells (hiPSCs). In contrast, no significant difference in the expression of EpCAM was observed between differentiated and undifferentiated cells (Figure 1B,C).

Next, barrier integrity and subcellular localization was evaluated using immunocytochemical staining for the cell adhesion endothelial protein PECAM, as well as the tight junction-associated proteins occludin and ZO-1 (Zonula Occludens-1) (Figure 1D). The characteristic membrane localization and continuous junctional staining pattern observed for these proteins indicates the formation of tight cell–cell contacts in brain endothelial cells, which are essential for preventing paracellular transport of small molecules.

In addition, we assessed the expression of P-glycoprotein, an integral membrane protein that functions as an ATP-dependent efflux pump. As shown in Figure 1D, the staining pattern revealed a strikingly diffuse signal in intracellular compartments in addition to the expected membrane-associated localization. To assess the expression and localization of transport proteins relevant to BBB function and targeted nanoparticle uptake, we performed immunofluorescence staining for glucose transporter 1 (GLUT1) and the cation-independent mannose-6-phosphate receptor (CI-M6PR) in hiPSC-BMECs. As shown in Supplementary Figure S1, hiPSC-BMECs exhibited strong membrane-associated expression of GLUT1, a canonical marker of BBB endothelium responsible for facilitated glucose transport into the brain. Notably, we also observed clear intracellular and perinuclear staining of CI-M6PR, consistent with its known localization to endosomal and trans-Golgi networks [14,15]. These results confirm that our hiPSC-derived BMECs exhibit characteristic BBB transporter expression profiles.

Successful differentiation of hiPSC-BMECs is typically characterized by a high transendothelial electrical resistance (TEER) and low paracellular permeability. Accordingly, we measured TEER throughout the differentiation process and observed a progressive increase, reaching approximately 4000 Ω·cm^2^ by day 10 (Figure 1E). Early differentiation protocols, such as those by Lippmann et al. (2012) [20], achieved TEER values in the range of 300–400 Ω·cm^2^, which were initially considered a significant advance for in vitro BBB modeling. However, more recent refinements, including the incorporation of Wnt pathway activation (e.g., via CHIR99021) and improved media formulations, have led to reports of TEER values exceeding 4000–5000 Ω·cm^2^, particularly when using defined extracellular matrices and optimized co-culture systems [29,31,32,33]. The TEER values observed in our system indicate the successful formation of a high-resistance endothelial barrier and suggest effective induction of BBB-specific phenotypes, therefore the experiments were performed at day 10 of differentiation.

High molecular weight dextrans are commonly used as paracellular permeability markers due to their inability to cross intact BBBs under normal conditions. We assessed the permeability of FITC-labeled dextran (70 KDa) over a 6 h period using hiPSC-BMECs cultured on 3 µm pore transwell inserts [34]. As shown in Figure 1F, only ~0.3% of dextran was detected in the basolateral compartment, with no appreciable increase over time, indicating minimal paracellular leakage.

In contrast, transferrin-mediated transcytosis across the BBB is a well-characterized and widely studied mechanism for CNS drug delivery [8,9,10,13,35]. In our experimental design, we used transferrin (Tfr) conjugated to a Cy3 fluorophore as a positive control for receptor-mediated transcytosis. As expected, Tfr-Cy3 transport increased over time (Figure 1F), confirming that our hiPSC-BMEC model recapitulates key aspects of BBB selective permeability and active transport mechanisms.

Although the present hiPSC-derived BBB model recapitulates key endothelial features of the human BBB, including high TEER values and low paracellular permeability, it remains an in vitro system that cannot fully reproduce the structural and physiological complexity of the neurovascular unit in vivo.

### 3.2. Liposomes Manufacture and Characterization

**Figure 2:**
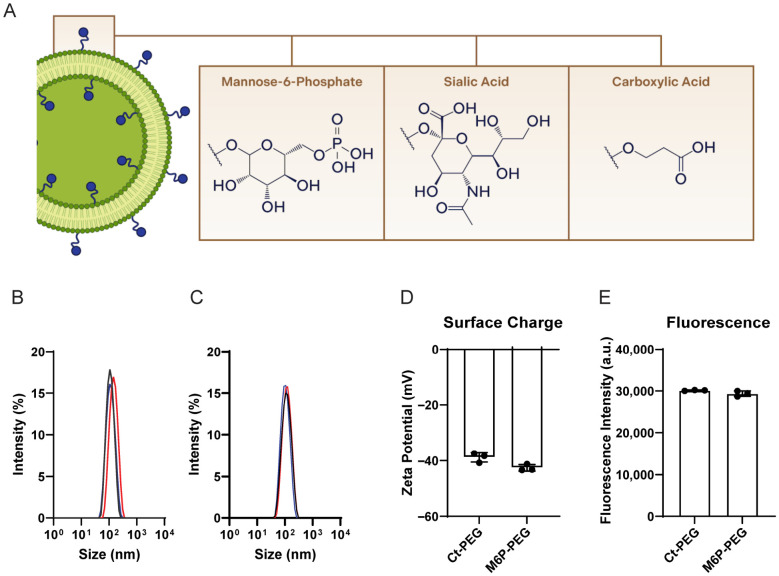
Physicochemical characterization of Ct and M6P liposomes. (**A**) Scheme of the liposomes used in this study, showing the different ligands attached to the end point of the PEG chain. (**B**) Ct (40 mol% cholesterol, 34.5 mol% DOPC, 0.5 mol% DiO, 15 mol% DSPE-PEG8-OCH3) and (**C**) M6P (40 mol% cholesterol, 34.5 mol% DOPC, 0.5 mol% DiO, 15 mol% DSPE-PEG8-M6P) size distribution of prepared liposomes. Each color curve represents one independent liposome preparation. (**D**) The surface charge determined as zeta potential of both formulations shows values between −35 and −45 mV. (**E**) Absolute DiO fluorescence of two representative Ct and M6P liposome formulations (100 µM total lipid concentration) used for quantitative comparison in the biological experiments. Each data point represents one liposome independent preparation.

To evaluate the influence of surface functionalization on liposome interaction with the blood–brain barrier (BBB), we developed a series of liposomal formulations (Figure 2A) labeled using the lipophilic fluorescent tracer DiO or DiD. All formulations were prepared to maintain a uniform size with a diameter within the range of 90–130 nm and the polydispersity index (PDI) values consistently below 0.2, indicating a homogenous size distribution. This ensured that any observed differences in biological behavior could be attributed to surface modifications rather than variations in particle size or homogeneity.

Previously, we demonstrated that M6P ligand on the surface of liposomes enhances liposomal uptake in various cell lines [25]. Therefore, based on the previous reports of M6P involvement in transcytosis of viruses, we hypothesized that the M6P-PEG-lipid conjugates could also play a role as a putative transcytosis enhancer in the liposomal formulations. We synthesized DSPE-PEG-mannose-6-phosphate (M6P) conjugates and incorporated them into the liposomal formulations to explore how M6P ligands influence liposome-cell interactions. The chemical structures of the used ligands are shown in the Supplementary Figure S2. The DSPE-PEG-ligand conjugates were synthesized via strain-promoted acyl-alkyl cycloaddition (SPAAC), a copper-free click chemistry approach known for its bioorthogonality and efficiency under mild conditions. The synthetic methodology followed previously established protocols from our laboratory, enabling precise and reproducible ligand synthesis and incorporation into the liposome formulations [25].

The liposomes were prepared using thin-film hydration method, followed by liposome sonication and extrusion to optimize the size of the resulting vesicles. As the ligands were included in the lipid composition right from the beginning, we expect the ligands to be present in both the inner and the outer phospholipid bilayers. Both control DSPE-PEG (Ct) and DSPE-PEG-mannose-6-phosphate (M6P) liposomes showed a similar size distribution with a mean around 110 nm for both formulations (Figure 2B,C) and PDI values are in both case lower than 0.2, indicating a narrow size distribution range. These formulations exhibited a net negative surface charge, with zeta potential values around −40 mV (Figure 2D). The net fluorescence values from both formulations did not show significant differences (Figure 2E), allowing for quantitative comparisons of liposome uptake using fluorescence measurements as a readout.

As further controls, we employed alternative DSPE-PEG conjugates bearing structurally distinct terminal moieties, including sialic acid and carboxylic acid (Supplementary Figure S3), and the ganglioside GM1 (Supplementary Figure S4), which is known to interact with raft-mediated uptake pathways and certain lectins [36,37]. These formulations exhibited a net negative surface charge, with zeta potential values ranging between −20 and −40 mV (Supplementary Figures S3 and S4), depending on the nature and density of the conjugated ligands.

### 3.3. M6P Ligand Increases the Uptake of Liposomes by hiPSC-Derived BMECs

**Figure 3:**
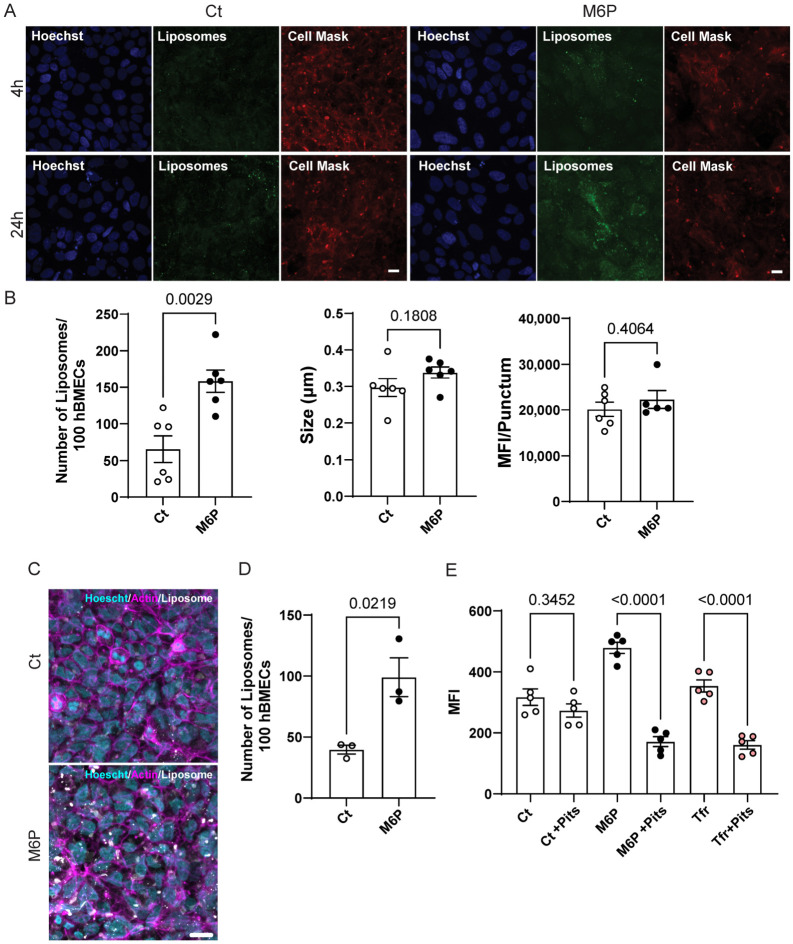
M6P-functionalization enhances liposome uptake in hiPSC-BMECs via clathrin-mediated endocytosis. (**A**) Confocal microscopy images showing internalization of DiO-labeled functionalized liposomes with either control-PEG (Ct) or 15% mannose-6-phosphate-PEG (M6P) in hiPSC-BMECs after 4 and 24 h of treatment (100 µM total lipid concentration). Hoechst stains nuclei, CellMask outlines cell membranes, and DiO fluorescence indicates liposomes. Bar = 10 µm. (**B**) Quantification of confocal images after 4 h treatment shows significantly higher liposome uptake in the M6P treated cells compared to the Ct treated cells. (**Left**: number of liposomes per 100 cells, *p* = 0.0029; **middle**: average puncta size; **right**: mean fluorescence intensity (MFI) per punctum). Each data point represents one well from an independent differentiation culture. Data are shown as mean ± s.e.m.; statistical significance is indicated, calculated by Student’s *t*-test. (**C**) Representative confocal images of liposomes uptake (500 µM total lipid concentration, 2 h) of 15% M6P compared to Ct. Bar = 10 µm. (**D**) Images quantification of internalized liposomes (500 µM) shows increased M6P uptake over Ct at 2 h treatment. Each data point represents one well from an independent differentiation culture. Data are shown as mean ± s.e.m.; statistical significance is indicated, calculated by Student’s *t*-test. (**E**) Flow cytometry analysis showing the effect of Pitstop 2 (Pits), a clathrin-mediated endocytosis inhibitor, in liposomes and Tfr internalization. Each data point represents one well from an independent differentiation culture. Data are shown as mean ± s.e.m.; statistical significance is indicated, calculated by one-way ANOVA and Tukey post hoc test.

To assess the impact of M6P ligand conjugation on liposome fluorescence-based uptake in hiPSC-BMECs, we compared the internalization of M6P-functionalized liposomes (M6P) versus non-targeted liposomes (Ct) (Figure 3A). HiPSC-BMECs were treated with 100 µM total lipid concentration of either Ct or M6P liposomes for 4 or 24 h. In cells treated with Ct (visualized via DiO fluorescence), the internalized liposomes were almost undetectable at both 4 and 24 h. In contrast, cells treated with M6P liposomes displayed a punctate intracellular fluorescence pattern at 4 h. This pattern increased in intensity over time and became more diffuse by 24 h, suggesting progressive internalization and potential diffusion of the dye out of the liposomes upon their degradation.

As shown in Figure 3B, quantitative fluorescence analysis after 4 h of incubation revealed a significant increase in the cellular uptake of M6P compared to Ct treatment. This enhancement was consistent across multiple biological replicates, corroborating our previous observations that the presence of the M6P ligand on the nanoparticle surface contributes to increased cellular uptake, potentially through ligand-dependent internalization pathways [25]. As expected, when the size of the puncta and the mean fluorescence intensity (MFI) of individual puncta were analyzed, no significant differences were observed between the two treatments (Figure 3B).

Next, we investigated the involvement of clathrin-dependent endocytosis in the uptake of liposomes. Pitstop 2 (Pits) is a selective, cell-permeable inhibitor of clathrin-mediated endocytosis (CME) that functions by binding to the terminal domain of clathrin [38]. Due to observed cytotoxicity at longer incubation times with Pits, we selected a 2 h time point for further analysis. After 2 h of incubation of hiPSC-BMECs with 500 µM total lipid concentration liposomes without an inhibitor, we observed enhanced internalization of M6P compared to Ct when we quantified the number of liposomes inside the cells using microscopy (Figure 3C,D), which allowed us to proceed with this time point for the inhibition studies.

For the inhibition studies, the cells were preincubated for 15 min with either 25 µM Pits or vehicle control, followed by treatment with 500 µM total lipid concentration liposomes of either Ct or M6P for 2 h. Internalization was then assessed by flow cytometry. As shown in Figure 3E, Pits significantly reduced the internalization of both M6P liposomes and the positive control transferrin (Tfr), indicating a role for CME in their uptake. In contrast, the internalization of Ct was not affected by Pits treatment. These results support the hypothesis that M6P-mediated uptake of liposomes mainly occurs via clathrin-dependent endocytosis in hiPSC-BMECs, while non-functionalized liposomes may use other less specific uptake pathways. Although CI-M6PR is a biologically plausible mediator of the observed M6P effect, our current data do not establish CI-M6PR-specific causality; future work should test this directly through free M6P or IGF2 competition, receptor-blocking antibodies, or CI-M6PR knockdown/knockout.

### 3.4. M6P Functionalization Enhances Transcytosis of Liposomes Across hiPSC-Derived BMECs

**Figure 4:**
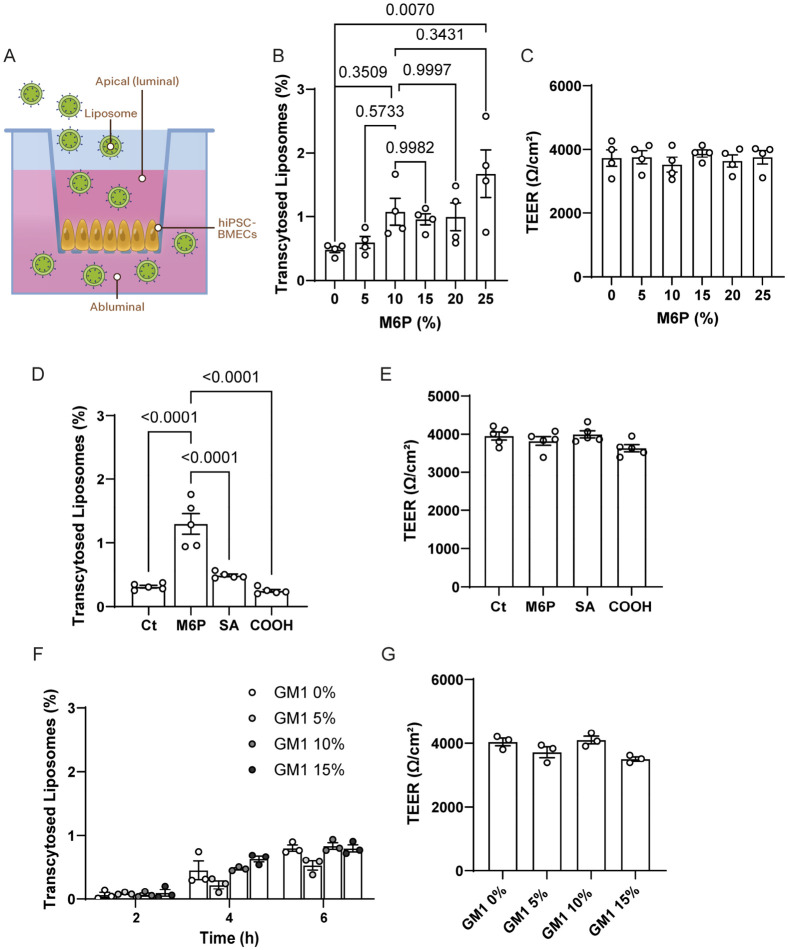
M6P functionalization enhances liposomes transcytosis across hiPSC-BMECs. (**A**) Schematic of the transcytosis assay setup using hiPSC-derived brain microvascular endothelial cells (hiPSC-BMECs) cultured on 3 µm pore transwell inserts. Liposomes were applied to the apical side, and abluminal fluorescence was quantified during or after 6 h. (**B**) Transcytosis efficiency of liposomes (40 mol% cholesterol, 34.5 mol% DOPC, 0.5 mol% DiO, 0–25 mol% DSPE-PEG8-OCH3 and 0–25 mol% of DSPE-PEG8-M6P) with increasing molar percentages (0–25 mol%) of M6P, measured by DiO fluorescence in the abluminal compartment. A dose-dependent increase in transcytosis was observed, with a plateau at ~10% mol M6P, indicating enhanced transport efficiency via M6P ligand–receptor interactions. Each data point represents one transwell from an independent differentiation culture. (**C**) TEER values at the end of the experiment, showing the integrity of the barrier along the experiment. (**D**) Comparison of M6P liposomes to structurally similar liposomes control (40 mol% cholesterol, 44.5 mol% DOPC, 0.5 mol% DiO, 15 mol% DSPE-PEG8-OCH3, DSPE-PEG8-M6P, DSPE-PEG 8-SA, or DSPE-PEG8-COOH): Ct-PEG (Ct), sialic acid-PEG (SA), and carboxylic acid-PEG (COOH). Only M6P liposomes showed significantly increased transcytosis (*p* < 0.0001). Each data point represents one transwell from an independent differentiation culture. (**E**) Corresponding TEER values at the end of the experiment, showing the integrity of the barrier along the experiment. (**F**) Evaluation of liposomes formulated with increasing GM1 content (0–15% mol) showed a modest, non-significant increase in transcytosis. Each data point represents one transwell from an independent differentiation culture. (**G**) TEER values at the end of the experiment, showing the integrity of the barrier along the experiment. Data are presented as mean ± s.e.m. percentage of transported liposomes relative to initial fluorescence in apical compartment; statistical significance is indicated, calculated by one-way ANOVA and Tukey post hoc test.

Liposomes functionalized with mannose-6-phosphate ligand have demonstrated enhanced uptake efficiency correlating positively with the molar percentage of M6P on the liposome surface in cell lines [25]. To investigate whether this correlation extends to transcytosis across an in vitro model of the blood–brain barrier (BBB) (Figure 4A), we evaluated the effect of varying M6P surface densities on liposomes (Figure 4B) in transcytosis using an in vitro model comprising hiPSC-BMECs cultured on 3 µm pore transwell inserts (Figure 4A). The pore size of 3 µm was selected to minimize the hindrance of the liposome passage through interactions solely with the membrane, which we observed when working with a smaller pore size. Liposomes displaying surface M6P densities from 0 to 25 mol% were manufactured, characterized (Supplementary Figure S3), and applied to the apical (luminal) side at a total lipid concentration of 100 µM. After 6 h, the basolateral (abluminal) medium was collected, and transported particles were quantified via DiO fluorescence.

As depicted in Figure 4B, transcytosis efficiency increased with higher M6P surface content, reaching a plateau at approximately 10 mol% and higher mean values observed at 25 mol%, and it did not represent a statistically superior performance over the 10 mol% formulation. This suggests that M6P density on the liposome surface plays a crucial role in enhancing transcytosis, likely by increasing avidity at the cell surface and facilitating interaction with intracellular M6P receptors, including those in endo-lysosomal compartments, thereby supporting the completion of the transcytosis pathway (Supplementary Figure S1) [15]. To confirm that the transcytosis experiments did not compromise endothelial barrier integrity, TEER values were measured at the end of each experiment. No significant differences in TEER were observed between treatment groups and control conditions, indicating that exposure to M6P-functionalized or control liposomes did not disrupt the integrity of the hiPSC-BMEC monolayer. These results support that the observed differences in liposome transport were not due to nonspecific barrier leakage or monolayer destabilization (Figure 4C).

To further assess the specificity of M6P-mediated transcytosis, we manufactured and characterized liposomes (Supplementary Figure S4) containing several structurally similar control ligands, including a sialic acid-PEG-lipid conjugate (to control for nonspecific monosaccharide binding) and a carboxylic acid-PEG-lipid conjugate (to control for general anionic interactions). All liposomes were applied to the apical side at a total lipid concentration of 100 µM, and DiO fluorescence in the basolateral compartment was measured after 6 h. As shown in Figure 4D, M6P demonstrated significantly higher transcytosis compared to all control formulations. Importantly, transendothelial electrical resistance (TEER) values remained stable throughout the assay, indicating preserved barrier integrity and ruling out paracellular leakage as a confounding factor (Figure 4E).

To further explore the influence of glycan structures on liposome transcytosis, we investigated the effect of increasing ganglioside GM1 content on transport across the hiPSC-BMEC barrier (Figure 4F). GM1 is a sialylated glycosphingolipid naturally enriched in neuronal membranes and is known to engage with various lectins and lipid raft domains involved in endocytosis and intracellular trafficking [36,37,39]. Moreover, GM1 has been reported to interact with components of the endocytic machinery, including caveolin-1 and galectins, which may facilitate vesicular transport across endothelial barriers [39,40,41].

In this experiment, liposomes were formulated with increasing molar percentages of GM1 (ranging from 0 to 15 mol%), characterized (Supplementary Figure S5), and applied to the apical side of the transwell at a total lipid concentration of 100 µM. After 6 h, DiO fluorescence in the basolateral compartment was quantified. As shown in Figure 4F, a non-significant increase in transcytosis was observed with increasing GM1 content at all analyzed times, while after 6 h, TEER values remained stable throughout the assay, indicating preserved barrier integrity (Figure 4G). Although the general enhancement was less pronounced than that observed with M6P functionalization, these findings indicate that GM1 incorporation may offer a modest improvement in transcytosis, possibly by engaging endogenous glycan-recognition mechanisms or raft-associated trafficking pathways [42,43]. The inclusion of GM1 and SA as a comparator is particularly informative, as it enables discrimination between general glycan-mediated uptake.

Collectively, these findings confirm that the M6P-functionalized liposomes undergo ligand-dependent, M6P-specific, clathrin-sensitive uptake and enhanced transcytosis across the hiPSC-BMEC BBB model. However, the current data do not definitively establish CI-M6PR-specific causality. Future studies employing receptor competition, blocking antibodies, or genetic interference approaches will be necessary to define the relative contribution of CI-M6PR to the observed transcytosis process.

### 3.5. Efficient Transcytosis of Intact M6P Liposomes Across hiPSC-BMECs

**Figure 5:**
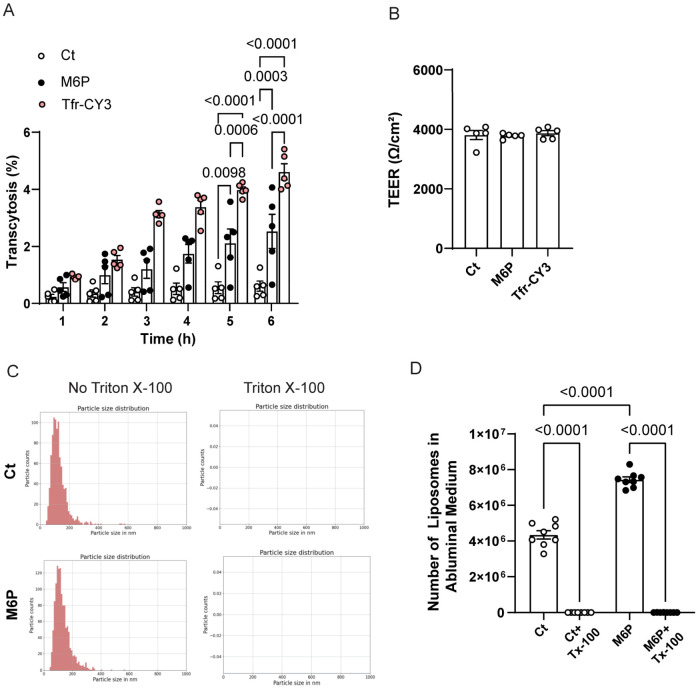
Intact M6P-functionalized liposomes transcytosed through hiPSC-BMECs. (**A**) Time-course quantification of liposome transcytosis across hiPSC-BMEC monolayers. It can be seen that 15% M6P liposomes showed significantly higher transport compared to Ct liposomes (*p* < 0.0001). Transferrin-Cy3 (Tfr-Cy3) served as a positive control for receptor-mediated transcytosis. Each data point represents one transwell from an independent differentiation culture. Data are presented as mean ± s.e.m.; statistical significance is indicated, calculated by one-way ANOVA and Tukey post hoc test. (**B**) TEER values after 6 h experiment, showing the integrity of the barrier along the experiment. (**C**) A representative histogram of nanoparticle tracking analysis (NTA) confirms the presence of intact liposomes in the abluminal compartment. Post-treatment with 0.1% Triton X-100, which disrupts the lipid bilayer and eliminated detectable fluorescence, verifying that the measured signal was associated with intact nanoparticles. (**D**) Quantification of liposomes amount in abluminal medium after 6 h transcytosis assay. Each data point represents one transwell from an independent differentiation culture. Data are presented as mean ± s.e.m.; statistical significance is indicated, calculated by one-way ANOVA and Tukey post hoc test.

As shown in Figure 5A, the time-course analysis of liposomes transcytosis revealed a significant and progressive increase in the percentage of M6P liposomes transported across the hiPSC-BMECs barrier over a 6 h period, with significantly higher levels than Ct at all time points. Transferrin-Cy3, used as a positive control, also exhibited efficient transport, validating the functionality of the in vitro BBB model. Importantly, the enhanced transcytosis observed in our hiPSC-BMEC model occurred without compromising barrier integrity, as demonstrated by stable TEER values (Figure 5B).

Transferrin is widely regarded as a gold standard for receptor-mediated transcytosis across the blood–brain barrier owing to its high-affinity interaction with the transferrin receptor, which is abundantly expressed on brain endothelial cells [8,9,10,44]. In this study, M6P-functionalized lipid nanoparticles demonstrated a transcytosis efficiency reaching approximately 55% of that observed for transferrin after incubation for 6 h (M6P 2.53 ± 0.59 compared to Tfr 4.61 ± 0.28). This is a promising achievement, as most ligand-functionalized nanoparticles described in the literature typically achieve only 10–30% of Tfr transcytosis under comparable in vitro conditions [9,10]. However, this comparison should be interpreted as an internal benchmark rather than a direct equivalence, since transferrin-Cy3 is a soluble ligand whereas M6P-liposomes are nanoscale vesicles whose transport depends on additional physicochemical and trafficking parameters.

A topic of active investigation and debate is whether intact liposomes or functionalized lipid nanoparticles can cross in vitro models of the blood–brain barrier. To address this, and to verify that the fluorescence detected in the abluminal medium was originated from intact liposomes, we treated the medium collected from hiPSC-BMEC incubated with Ct or M6P liposomes for 6 h with 0.1% Triton X-100, a non-ionic detergent that solubilizes lipid membranes. Fluorescent particles were then quantified using nanoparticle tracking analysis (NTA). As shown in Figure 5C, we observed intact fluorescent liposomes with an average size around 100 nm in the abluminal medium, and following Triton X-100 treatment, fluorescent liposomes were no longer detectable in either condition, confirming that the fluorescence signal in the abluminal medium was associated with intact lipid nanoparticles rather than free dye or degradation products. Moreover, when we quantified the number of liposomes in the abluminal medium, we observed a higher amount in M6P-treated cells compared to Ct. In both cases, the fluorescence signal was completely abolished in the presence of detergent (Figure 5D). These results provide strong evidence that intact lipid nanoparticles (and not free dye or degraded fragments) can cross in vitro BBB models. The complete loss of fluorescence signal after Triton X-100 treatment confirms that the detected signal in the abluminal compartment originated from membrane-enclosed particles. Furthermore, the higher number of liposomes observed with M6P functionalization compared to controls supports the role of M6P as an effective ligand to enhance transcytosis across brain endothelial cells.

### 3.6. M6P-Functionalized Liposomes Exhibit Enhanced Uptake by Astrocytoma Cells and hiPSC-Derived Neurons Following Transcytosis

**Figure 6:**
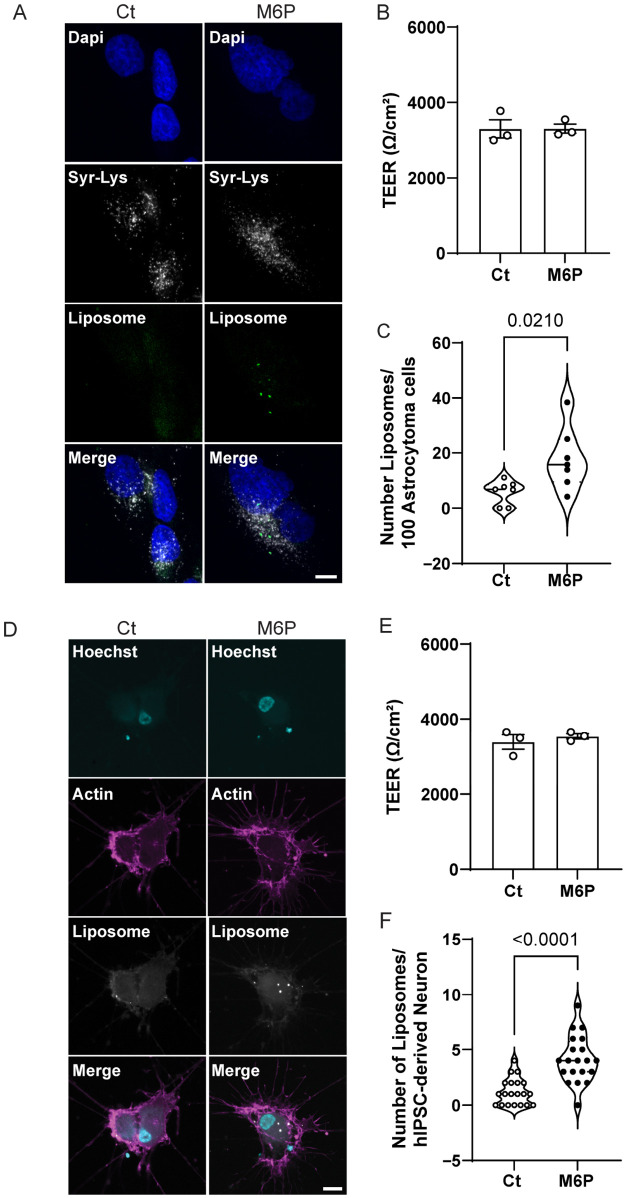
M6P-functionalized liposomes exhibit enhanced uptake by downstream astrocytoma cells and hiPSC-derived neurons. (**A**) Immunofluorescence images of astrocytoma cells located in the abluminal chamber after 6 h transcytosis assay, showing nuclei (Dapi, blue), Lysosomes (Syr-Lys, white) and liposome (DiO, green). It can be seen that 15% M6P liposomes accumulated in puncta in close proximity to lysosomes, suggesting intracellular uptake. Bar = 5 µm. (**B**) TEER values after 6 h experiment, showing the integrity of the barrier along the experiment. (**C**) Quantification of liposomes accumulation in downstream astrocytoma cells showed significant uptake of M6P liposomes versus Ct (*p* = 0.0210). Each data point represents one biological independent culture. Data are shown as mean ± s.e.m.; statistical significance is indicated, calculated by Student’s *t*-test. (**D**) Representative confocal images of abluminal seeded hiPSC-derived neurons after transcytosis assay, showing nuclei (Hoechsti, cyan), Actin (Syr-Actin, magenta) and liposome (DiD, white). Neurons showed greater internalization of 15% M6P liposomes (DiD, white puncta) than Ct, with a vesicular distribution pattern. Bar = 10 µm. (**E**) TEER values after 6 h experiment, showing the integrity of the barrier along the experiment. (**F**) Quantitative analysis of neuronal liposomes uptake revealed significantly higher numbers of M6P particles per neuron compared to Ct (*p* < 0.0001). Each data point represents one neuron from independent differentiation culture. Data are shown as mean ± s.e.m.; statistical significance is indicated, calculated by Mann–Whitney test.

To assess whether M6P-mediated transcytosis results in effective delivery of liposomes to downstream cells, we quantified the accumulation of M6P-functionalized liposomes compared to control liposomes in astrocytoma cells and hiPSC-derived neurons following transcytosis through a hiPSC-BMEC monolayer. In this Transwell system, the hiPSC-BMECs were seeded on the upper (luminal) side of the porous membrane to form a tight monolayer mimicking the blood–brain barrier. Neurons and astrocytoma cells were cultured in the lower (abluminal) chamber, beneath the endothelial barrier, to simulate the brain parenchyma environment. After 6 h of incubation with liposomes added to the luminal compartment, we quantified the amount of liposome-associated fluorescence internalized by the cells in the lower chamber (Figure 6). This setup allowed us to evaluate not only the ability of M6P-functionalized liposomes to cross the endothelial barrier but also their subsequent uptake by brain-relevant target cells. To confirm that the transcytosis assay did not compromise barrier integrity, TEER measurements were performed at the end of the experiment, and no differences were observed between treatment conditions, indicating preservation of the hiPSC-BMEC monolayer integrity throughout the assay (Figure 6B).

We first tested the intracellular localization of liposomes by immunofluorescence analysis on astrocytoma cells treated with transcytosed M6P or Ct liposomes. As shown in Figure 6A, M6P liposomes were present within cells and localized in close proximity to lysosomes, suggesting internalization into the target organelle. Analysis of liposome internalization in astrocytoma cells revealed an enhanced uptake of M6P, with a significant increase in liposome accumulation relative to Ct liposomes (Figure 6C).

Next, we investigated the uptake of liposomes by hiPSC-derived neurons as target cells following the transcytosis process. To validate successful neuronal differentiation, we first characterized the phenotype of our hiPSCs after 21 days of directed differentiation into forebrain neurons. Immunofluorescence analysis confirmed the expression of key neuronal markers, including the AMPA receptor subunit GluR1, the presynaptic protein Bassoon, and the dendritic marker MAP2 (Supplementary Figure S6A) [41,42].

Given that neurons are generally considered challenging targets for liposomal delivery due to their relatively low endocytic activity, we next evaluated the direct uptake of control (Ct) and M6P-functionalized (M6P) liposomes in hiPSC-derived neurons. Cells were incubated for 2 h with each formulation at a total lipid concentration of 100 µM. As shown in Supplementary Figure S6B,C, neurons exposed to M6P exhibited significantly higher uptake compared to those Ct treated (C: 1.2 ± 0.26; M6P: 4.15 ± 0.47; *p* < 0.0001).

To assess whether M6P-functionalization also facilitates effective delivery of liposomes after crossing the BBB, we performed a transcytosis assay using hiPSC-BMECs cultured on transwell inserts with either M6P or Ct liposomes added to the luminal side, hiPSC-derived neurons seeded in the basolateral compartment served as the recipient cells. After a 6 h incubation, we first confirmed that the treatments did not compromise barrier integrity by measuring TEER values at the end of the experiment. No differences were observed between treatment conditions, indicating preservation of hiPSC-BMEC monolayer integrity throughout the assay (Figure 6E).

Immunofluorescence microscopy revealed substantially greater liposome accumulation in neurons treated with transcytosed M6P compared to Ct (Figure 6D). The liposomes (white, DiD fluorescence) displayed a punctate cytoplasmic distribution, suggesting localization in endocytic vesicles. Quantitative analysis in the neuronal soma (Figure 6F) confirmed a significantly higher number of liposomes per neuron in the M6P group.

Together, these findings demonstrate that M6P-functionalization enhances liposome transcytosis across hiPSC-BMECs and increases downstream uptake by neuronal target cells in this in vitro BBB model. As a proof-of-concept study, these results support the potential of M6P as an alternative ligand strategy to improve CNS delivery of liposomal nanocarriers. However, further studies incorporating therapeutic cargos, functional readouts, and in vivo validation will be necessary to determine the translational applicability of this approach for CNS-targeted therapies.

## 4. Conclusions

The present study demonstrates that mannose-6-phosphate (M6P) functionalization significantly enhances the uptake and transcytosis of liposomes across a physiologically relevant in vitro blood–brain barrier (BBB) model, thereby offering a promising strategy for CNS-targeted delivery. Using hiPSC-derived human brain microvascular endothelial cells (hiPSC-BMECs), we developed a BBB model with robust barrier properties characterized by TEER values around 4000 Ω·cm^2^ and low paracellular permeability to 70 kDa dextran, surpassing earlier models using immortalized or primary cells [13,15]. These data confirm the effectiveness of Wnt/β-catenin activation via CHIR99021 in enhancing endothelial identity and BBB functionality, aligning with previous reports [22,24].

Surface functionalization of liposomes with M6P significantly increased cellular uptake in hiPSC-BMECs compared to control PEGylated liposomes, confirming prior findings in other cell types where M6P enhanced liposomal uptake via the clathrin-mediated pathway [18]. Clathrin-dependence of this uptake was substantiated by pharmacological inhibition with Pitstop 2, which markedly reduced M6P liposomes internalization but had minimal impact on control liposomes (Figure 3E), suggesting a relevant role for clathrin-dependent endocytic process.

Crucially, M6P conjugation also enhanced the transcytosis of liposomes across the BBB model. Transwell assays revealed a dose-dependent increase in liposome transport with increasing M6P ligand density, plateauing at ~10 mol% surface content (Figure 4B). This plateau suggests receptor saturation or trafficking bottlenecks, consistent with models of receptor-mediated transcytosis [30]. Compared to control ligands (sialic acid and carboxyl group), only M6P induced significant transcytosis, reinforcing ligand-specific receptor engagement. Transcytosed liposomes maintained their structural integrity, as verified by detergent disruption and nanoparticle tracking analysis (Figure 5C,D), indicating successful vesicular transport rather than passive diffusion or dye leakage.

Furthermore, the M6P-mediated enhancement in transcytosis translated into increased liposomes delivery to post-endothelial target cells. Astrocytoma cells and hiPSC-derived neurons located in the abluminal compartment accumulated significantly higher levels of M6P liposomes compared to controls, with intracellular localization suggesting endosomal or lysosomal processing. Importantly, M6P liposomes achieved transcytosis efficiencies of ~55% of that of transferrin-Cy3 (Figure 5A), a well-established ligand for receptor-mediated BBB transport [28,29]. This level of performance compares favorably with other ligand-targeted systems, which typically achieve 10–30% of transferrin efficiency under similar conditions [29]. Interestingly, the comparison with ganglioside GM1-functionalized liposomes revealed only modest effects on transcytosis (Figure 4F) despite GM1’s known role in raft-mediated endocytosis and neuronal targeting [31,36]. This underscores the superior specificity and efficacy of M6P as a ligand in crossing the BBB.

Our findings build upon our previous work demonstrating that M6P-functionalized liposomes exhibit enhanced cellular uptake across multiple cell types compared with structurally related control formulations [25]. In that study, M6P-mediated uptake was shown to be ligand-specific, as neither similarly anionic liposomes nor structurally related glycans reproduced the effect. Moreover, uptake of M6P was markedly reduced by Pitstop 2 treatment, supporting the involvement of a clathrin-associated endocytic pathway. Free M6P competition experiments also partially reduced liposome internalization, suggesting participation of an M6P-sensitive receptor pathway, although not definitively establishing CI-M6PR-specific causality. Together with the current data, these observations support the conclusion that M6P-functionalization promotes ligand-dependent and clathrin-sensitive uptake and transcytosis across hiPSC-derived BMECs. Importantly, the present study extends these earlier findings by demonstrating that enhanced uptake is associated with increased transport across a physiologically relevant human BBB model and improved downstream delivery to neurons and astrocytoma cells. Although CI-M6PR represents a biologically plausible mediator based on its known role in M6P trafficking and clathrin-associated internalization, the current data do not exclude the possibility that additional glycan-sensitive uptake mechanisms contribute to the observed phenotype. Future studies employing receptor-interference approaches, including free M6P or IGF2 competition, receptor-blocking antibodies, or CI-M6PR knockdown/knockout models, will therefore be important to establish receptor-specific causality more definitively (Table 1).

While the present study establishes M6P-functionalization as a strategy to enhance liposome uptake and transcytosis across a human in vitro BBB model, it does not yet demonstrate the delivery of a therapeutic cargo or functional rescue in downstream cells. Future studies should evaluate cargo-loaded M6P-liposomes, including small molecules, proteins/enzymes, or nucleic acids, and determine whether M6P decoration influences intracellular routing, cargo release, and bioavailability. Given the known role of M6P in lysosomal trafficking, this strategy may be particularly suitable for cargos intended for endosomal or lysosomal delivery, although it may require additional formulation optimization for cargos requiring cytosolic release. In vivo biodistribution, brain penetration, safety, and disease-model efficacy studies will be required before therapeutic relevance can be established.

## Figures and Tables

**Table 1:** Summary of the physicochemical characteristics and biological performance of control and M6P-functionalized liposomes in the hiPSC-derived BBB model. The table summarizes liposome composition, ligand density, particle size, polydispersity index (PDI), zeta potential, uptake behavior, transcytosis efficiency, relative transport compared with transferrin, and downstream delivery to astrocytoma cells and hiPSC-derived neurons. Results highlight the enhanced uptake and BBB transcytosis achieved with M6P-functionalized liposomes relative to control formulations.

Parameter	Ct-PEG Liposomes	M6P-PEG Liposomes	Key Finding
Core composition	Cholesterol + DOPC + DSPE-PEG8-OCH3 + DiO/DiD	Cholesterol + DOPC + DSPE-PEG8-M6P + DiO/DiD	Same lipid backbone used for all formulations
Ligand	PEG only (control)	Mannose-6-phosphate (M6P)	Glycan-mediated targeting strategy
Particle size	108.7 ± 9.36 nm	116.2 ± 9.26 nm	Comparable nanoscale size
PDI	<0.2	<0.2	Homogeneous size distribution
Zeta potential	−38.89 ± 0.96 mV	−42.59 ± 0.67 mV	Similar negatively charged surface
Fluorescence normalization	No significant difference	No significant difference	Uptake comparisons not biased by fluorophore intensity
hiPSC-BMEC uptake (4 h)	65.67 ± 18.08	158.2 ± 15.43	Significant increase with M6P functionalization
Clathrin inhibition (Pitstop 2)	No significant effect	Strong reduction in uptake	Uptake is clathrin-sensitive
Ligand-density response	Not applicable	Increased transport from 5 to 25 mol% M6P	Plateau observed around ≥10 mol%
Transcytosis efficiency (6 h)	0.6 ± 0.18%	2.53 ± 0.59%	Enhanced BBB transport
Relative transport vs transferrin	~13% of Tfr-Cy3	~55% of Tfr-Cy3	Strong performance relative to BBB benchmark
Comparator ligands	SA-PEG, COOH-PEG, GM1	M6P-PEG	M6P showed highest transport specificity
Barrier integrity (TEER)	Preserved	Preserved	No monolayer disruption after treatment
Intact particle recovery (NTA)	Low particle recovery	Higher intact particle recovery	Transported particles remained structurally intact
Astrocytoma uptake after transcytosis	5.83 ± 1.6	17.86 ± 4.2	Increased downstream cellular delivery
hiPSC-neuron uptake after transcytosis	1.2 ± 0.26	4.15 ± 0.47	Improved neuronal delivery
Overall interpretation	Limited BBB transport	Enhanced uptake + transcytosis + downstream delivery	Proof-of-concept CNS delivery strategy using M6P-functionalized liposomes

## Data Availability

Dataset available on request from the authors.

## Supplementary Materials

The following supporting information can be downloaded at: https://www.mdpi.com/article/10.3390/pharmaceutics18050619/s1, Figures S1: Expression of key transporter and receptors in hiPSC-BMECs; Figures S2: Schematic representation of the synthesis of mannose-6-phosphate (M6P)-PEG-lipid conjugates and corresponding control ligands; Figures S3: Physicochemical characterization of control ligands carrying liposomes.; Figures S4: Physicochemical characterization of GM1-containing Liposomes; Figures S5: Physicochemical characterization of increasing M6P ligand-containing Liposomes; Figures S6: Characterization of liposomes uptake in iPSC-derived neurons.
