# Supplementary material for: Mannose-6-Phosphate-Tagged Liposomes Exhibit Increased Transcytosis Across Human Blood–Brain Barrier Model
Source: Pharmaceutics. 2026 May 19;18(5):619. doi: 10.3390/pharmaceutics18050619 (PMC13210468; doi:10.3390/pharmaceutics18050619)
Supplement: Supplementary file 1 [file pharmaceutics-18-00619-s001.zip › Supplementary Figure Legends.pdf]

Supplementary Figure Legends

Supplementary Figure S1: Expression of key transporter and receptors in hiPSC-BMECs. Representative images of immunofluorescence staining of hBMECs showing nuclei (Hoechst), actin (Phalloidin), CI-M6PR (cation-independent mannose-6-phosphate receptor), GLUT1 (glucose transporter 1), and merged image. Bar = 10 µm.

Supplementary Figure S2. Schematic representation of the synthesis of mannose-6-phosphate (M6P)-PEG-lipid conjugates and corresponding control ligands. The synthesized molecules consisted of a lipid anchor (DSPE) enabling incorporation into the liposomal membrane, a PEG-based linker, and the respective terminal ligand. Conjugates were synthesized using click chemistry reactions, yielding products with purities greater than 99%.

Supplementary Figure S3:Physicochemical characterization of control ligands carrying liposomes. (A) Size distribution, **(B)** surface charge, and **(C)** fluorescence of liposomes containing M6P and different control ligands. The formulations contain 15% total PEG, either as DSPE-PEG-M6P (M6P), or methoxy (Ct), sialic acid (SA), or carboxy (COOH) controls (40 mol% cholesterol, 44.5 mol% DOPC, 0.5 mol% DiO, 15 mol% DSPE-PEG8-OCH3, DSPE-PEG8-M6P, DSPE-PEG8-SA, or DSPE-PEG8-COOH). **(A)** The size of all prepared formulations is between 90 and 130 nm, with the PDI values below 0.2, confirming a narrow size distribution of the prepared liposomes. **(B)** All liposomes have negative zeta potential values ranging between -35 and -45 mV, regardless of the type of ligand used in the preparation. **(C)** The fluorescence of all formulations is in the same range between 27,000 and 28,000.

Supplementary Figure S4: Physicochemical characterization of GM1-containing Liposomes. (A) Size distribution, **(B)** surface charge, and **(C)** fluorescence of liposomes containing different amounts of GM1. The formulations contain 40 mol% cholesterol, 44.5 mol% DOPC, 0.5 mol% DiO, 0-15 mol% DSPE-PEG8-OCH3, and 15-0 mol% of GM1. **(A)** The size of all prepared formulations ranges between 90 and 130 nm, with the PDI values below 0.2, indicating a narrow size distribution of the prepared liposomes. **(B)** All liposomes have negative zeta potential values between -30 and -35 mV, regardless of the DSPE-PEG8-OCH3 and GM1 content. **(C)** The fluorescence of all formulations is in the same range between 32,000 and 33,000.

Supplementary Figure S5: Physicochemical characterization of increasing M6P ligand-containing Liposomes. (A) Size distribution, **(B)** surface charge, and **(C)** fluorescence of liposomes containing different amounts of DSPE-PEG8-M6P. The formulations contain 40 mol% cholesterol, 34.5 mol% DOPC, 0.5 mol% DiO, 0-25 mol% DSPE-PEG8-OCH3, and 25-0 mol% of DSPE-PEG8-M6P. **(A)** The size of all prepared formulations ranges between 90 and 130 nm, with the PDI values below 0.2, indicating a narrow size distribution of the prepared LNPs. **(B)** All liposomes have negative zeta potential values ranging between -35 and -45 mV, regardless of the DSPE-PEG8-OCH3 and DSPE-PEG8-M6P content. **(C)** The fluorescence of all formulations is in the same range between 31,000 and 33,000.

Supplementary Figure S6: Characterization of liposomes uptake in iPSC-derived neurons. (A) Representative immunofluorescence images of iPSC-derived neurons stained with GluR1 (Glutamate AMPA receptor subunit 1), Bassoon, and MAP2 to confirm neuronal identity. Bar = 10 µm. **(B)** Immunofluorescence images showing liposomes uptake in iPSC-derived neurons. Nuclei (DAPI), actin cytoskeleton (Syr-actin), and liposomes are shown in merged images. Bar = 10 µm. **(C)** Quantification of liposomes uptake per neuron following 2 h treatment with Ct-PEG and M6P-PEG formulations. Data are presented as mean ± SEM. Statistical significance was determined using an unpaired Student's t-test.
