# Supplementary material for: Mannose-6-Phosphate-Tagged Liposomes Exhibit Increased Transcytosis Across Human Blood–Brain Barrier Model
Source: Pharmaceutics. 2026 May 19;18(5):619. doi: 10.3390/pharmaceutics18050619 (PMC13210468; doi:10.3390/pharmaceutics18050619)
Supplement: Supplementary file 1 [file pharmaceutics-18-00619-s001.zip › Supplementary figure S6.pdf]

A

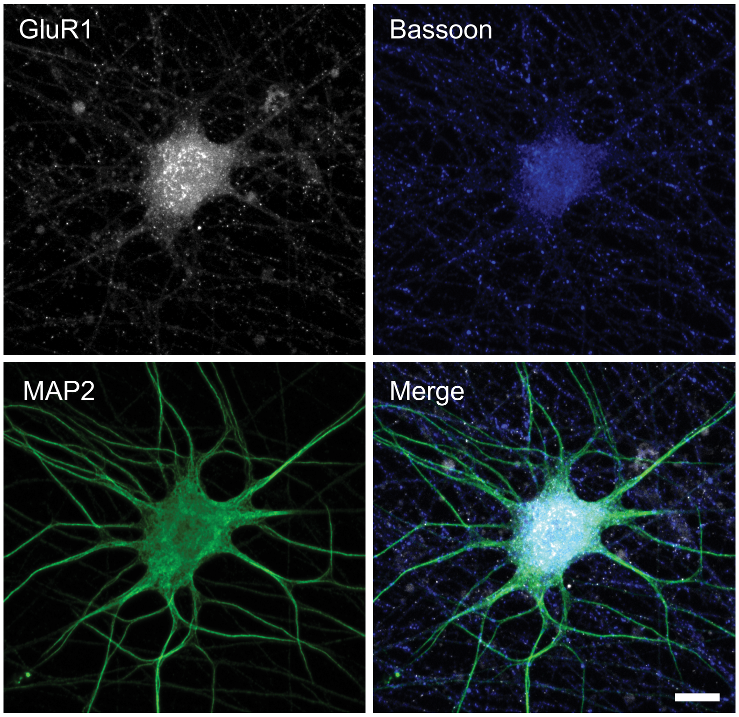
GluR1
Bassoon
MAP2
Merge

B

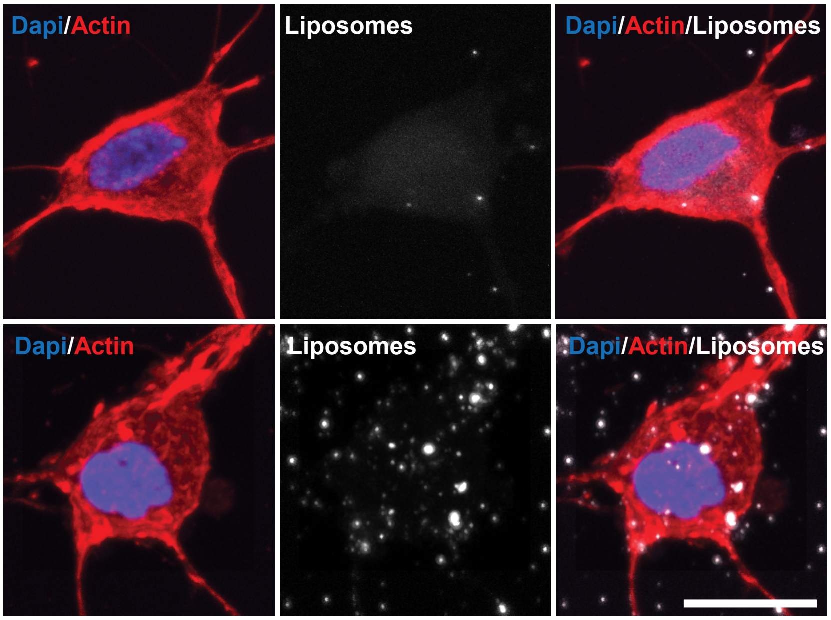
Dapi/Actin
Liposomes
Dapi/Actin/Liposomes
Dapi/Actin
Liposomes
Dapi/Actin/Liposomes

C

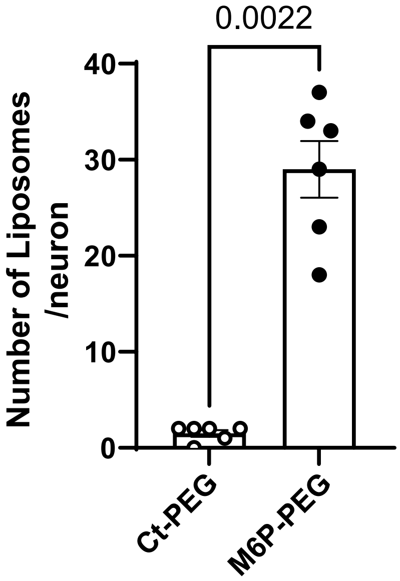
0.0022
40
30
20
10
0
Number of Liposomes /neuron
Ct-PEG
M6P-PEG
