# Supplementary figures and images for: Mannose-6-Phosphate-Tagged Liposomes Exhibit Increased Transcytosis Across Human Blood–Brain Barrier Model
Source: Pharmaceutics. 2026 May 19;18(5):619. doi: 10.3390/pharmaceutics18050619 (PMC13210468; doi:10.3390/pharmaceutics18050619)

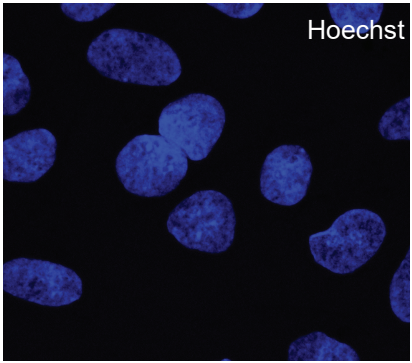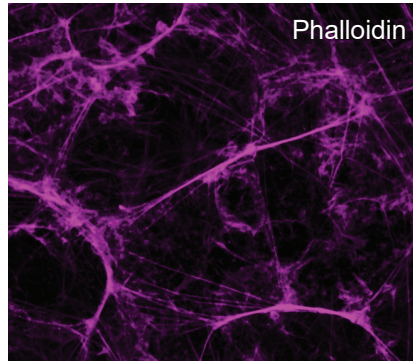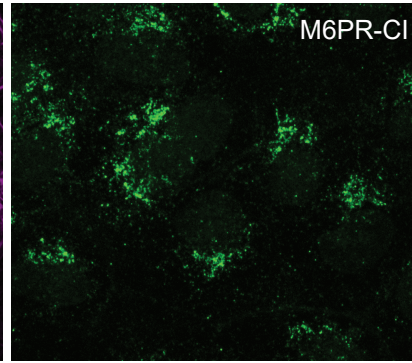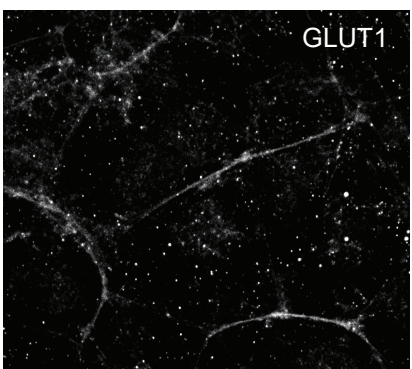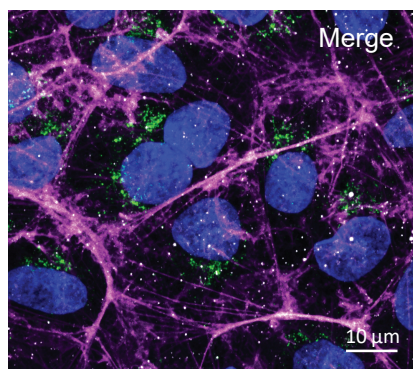

Supplement: Supplementary file 1 [file pharmaceutics-18-00619-s001.zip › Supplementary Figure S1.pdf]

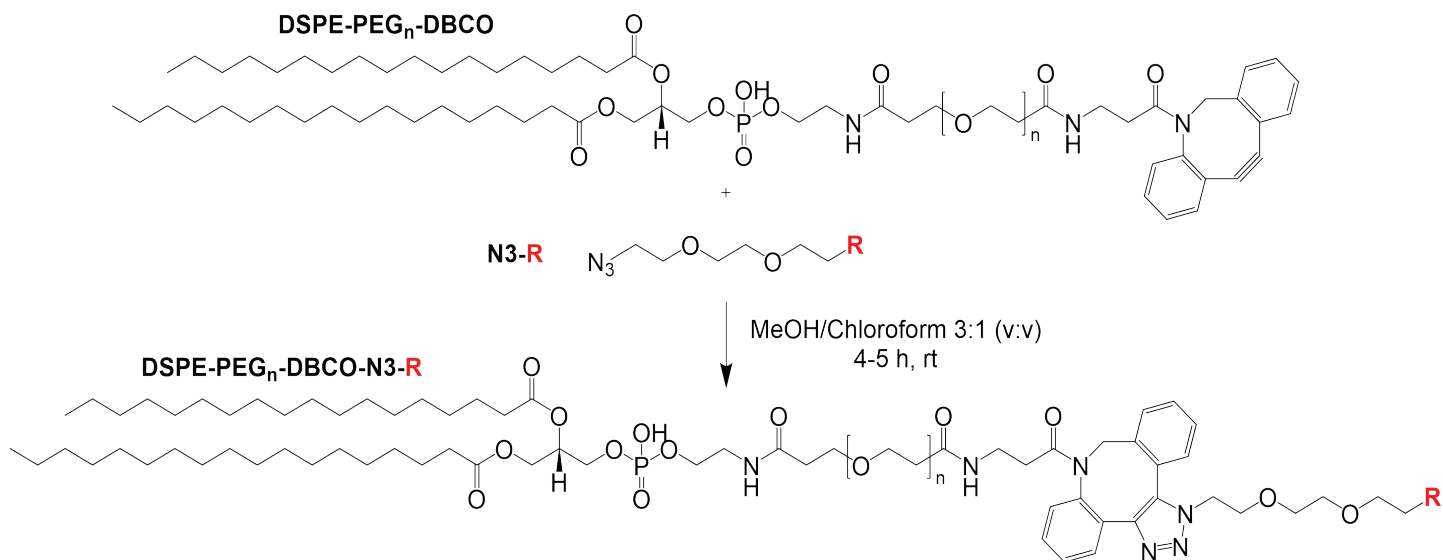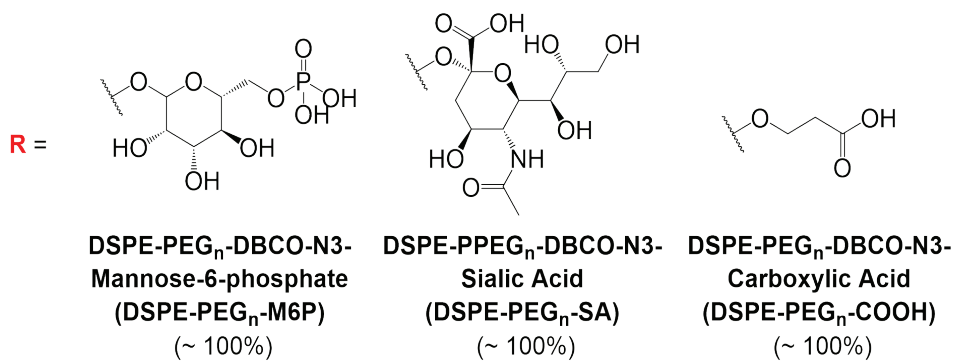

Supplement: Supplementary file 1 [file pharmaceutics-18-00619-s001.zip › Supplementary Figure S2.pdf]

A

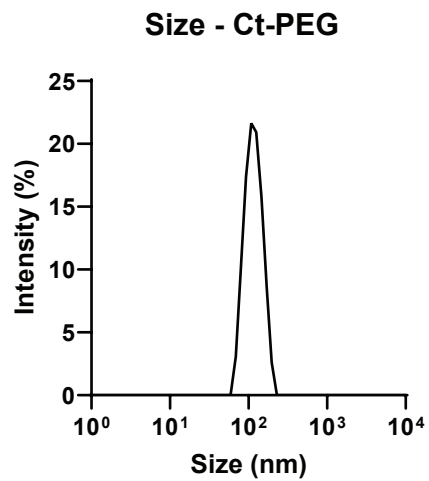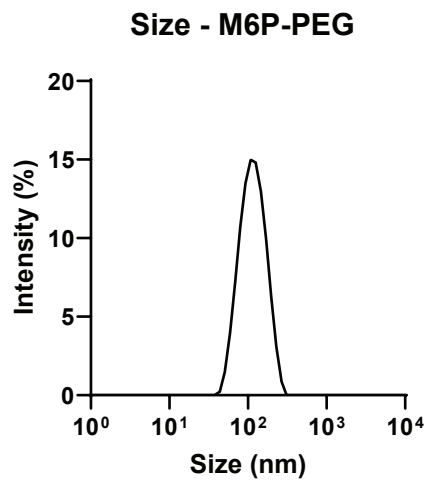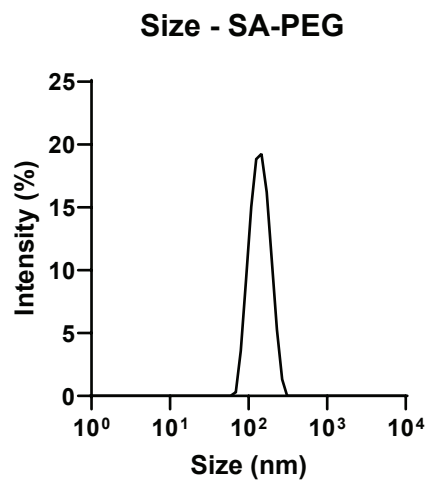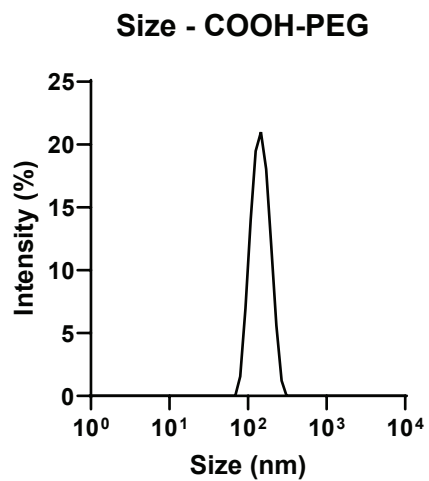

B

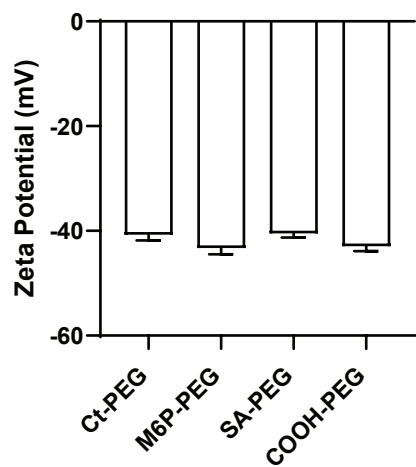

C

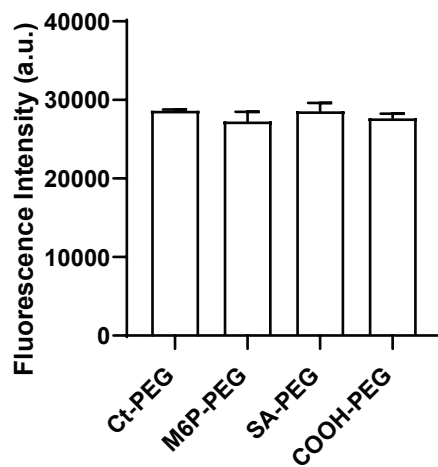

Supplement: Supplementary file 1 [file pharmaceutics-18-00619-s001.zip › Supplementary Figure S3.pdf]

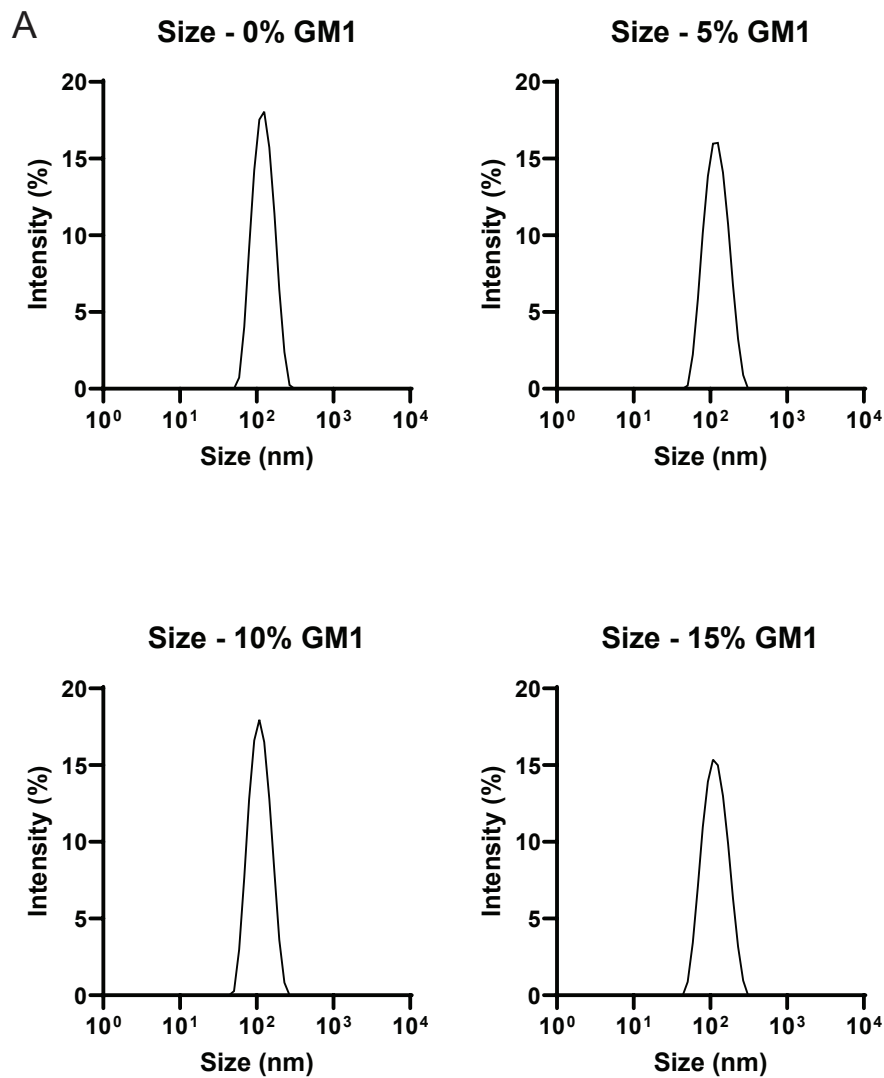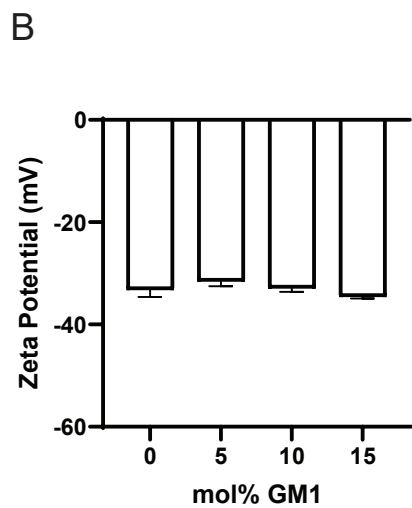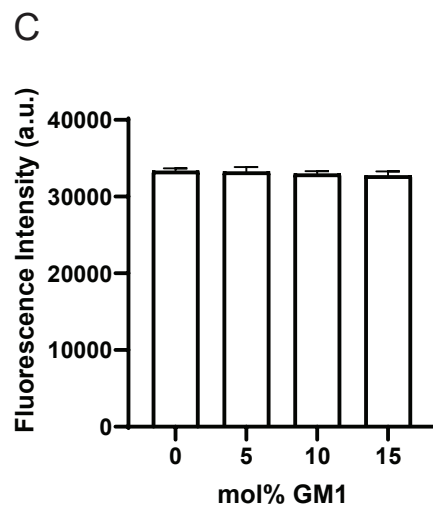

Supplement: Supplementary file 1 [file pharmaceutics-18-00619-s001.zip › Supplementary Figure S4.pdf]

A

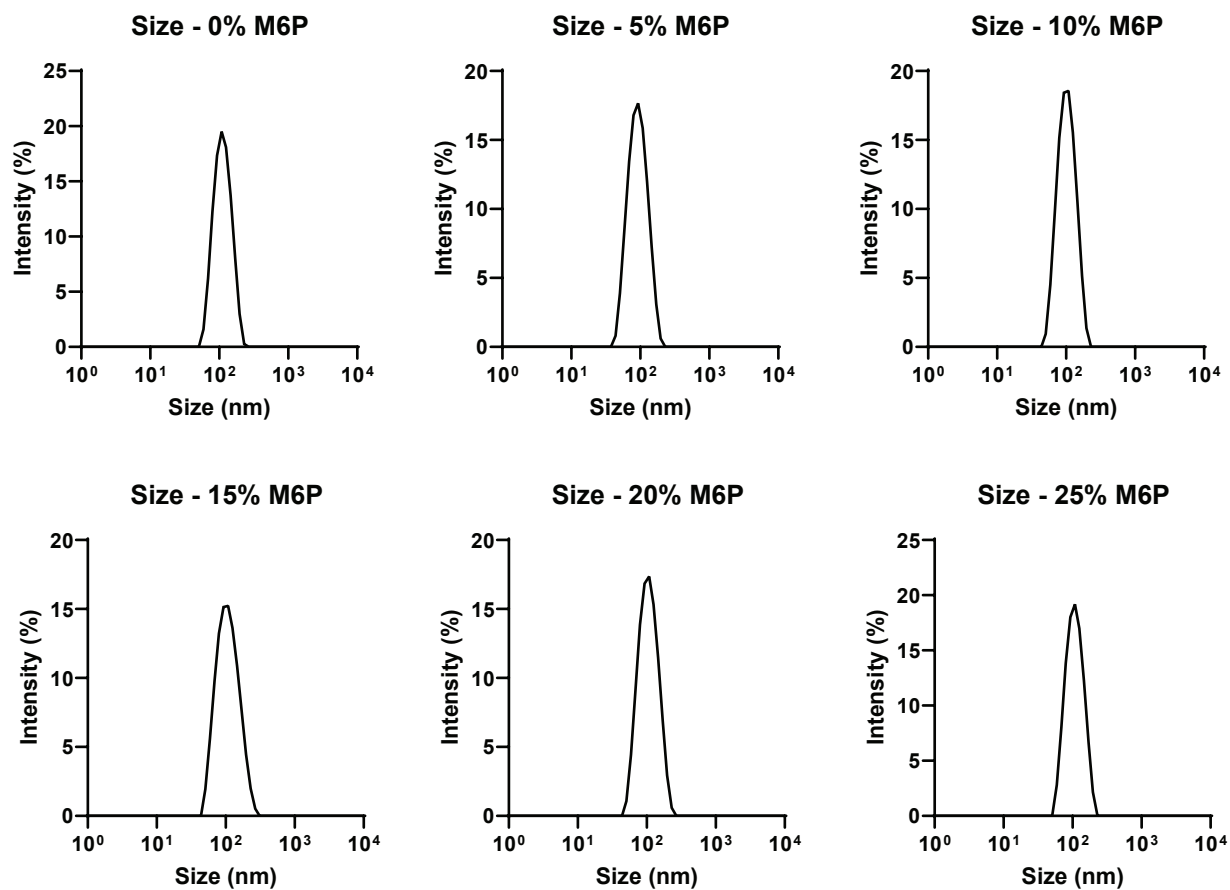

B

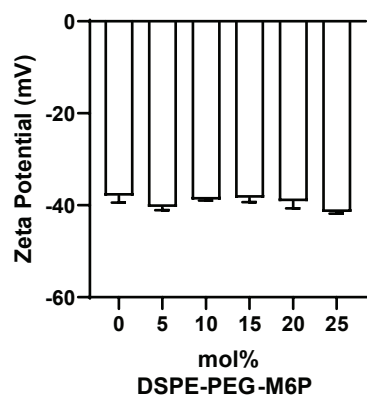

C

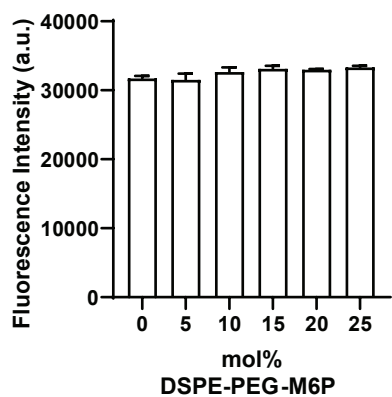

Supplement: Supplementary file 1 [file pharmaceutics-18-00619-s001.zip › Supplementary Figure S5.pdf]
